# Supplementary material for: Effects of DNA Origami‐Based Nanoagent Design on Apoptosis Induction in a Large 3D Cancer Spheroid Model
Source: Small. 2025 Apr 25;21(24):2502490. doi: 10.1002/smll.202502490 (PMC12177858; doi:10.1002/smll.202502490)
Supplement: Supplementary file 1 — Supporting Information [file SMLL-21-2502490-s001.docx]

**Supporting Information**

**Effects of DNA origami-based nanoagent design on apoptosis induction in a large 3D cancer spheroid model**

*Johann M. Weck, Riya Nair, Merve-Z. Kesici, Xiaoyue Shang,* *Svetozar Gavrilović, Cornelia Monzel, Amelie Heuer-Jungemann^*^*

J.M Weck, R. Nair, M.-Z. Kesici, S. Gavrilović, A. Heuer-Jungemann

Max Planck Institute of Biochemistry, Am Klopferspitz 18, 82152 Martinsried and Center for NanoScience (CeNS), Ludwig-Maximilians-University, Munich, Germany.

X. Shang, C. Monzel

Experimental Medical Physics, Heinrich-Heine University, Universitätsstraße 1, 40225 Düsseldorf, Germany.

Corresponding author: [heuer-jungemann@biochem.mpg.de](mailto:heuer-jungemann@biochem.mpg.de)

**Table of figures**

[**Figure S1: Design and simulation of the rro DNA origami** 4](#_Toc194070865)

[**Figure S2: Design and simulation of the mini DNA** **origami** 5](#_Toc194070866)

[**Figure S3: Design and simulation of the wf DNA origami** 6](#_Toc194070867)

[**Figure S4: Transmission electron microscopy (TEM) characterization of the rro DNA origami** 7](#_Toc194070868)

[**Figure S5: TEM characterization of the mini DNA origami** 8](#_Toc194070869)

[**Figure S6: TEM characterization of the wf DNA origami** 9](#_Toc194070870)

[**Figure S7: Cancer spheroid seeding and origami FISH** 10](#_Toc194070871)

[**Figure S8: Schematic of the clockscan protocol**  11](#_Toc194070872)

[**Figure S9: rro origami penetration through cancer spheroids** 12](#_Toc194070873)

[**Figure S10: mini origami penetration through spheroids** 13](#_Toc194070874)

[**Figure S11: wf origami penetration through spheroids** 14](#_Toc194070875)

[**Figure S12: Stability test of DNA origami** 15](#_Toc194070876)

[**Figure S13: TEM characterization of the rroOF nanoagents** 16](#_Toc194070877)

[**Figure S14: TEM characterization of the miniOF nanoagents** 17](#_Toc194070878)

[**Figure S15: TEM characterization of the wfOF nanoagents** 18](#_Toc194070879)

[**Figure S16: TEM characterization of the rroONF nanoagents** 19](#_Toc194070880)

[**Figure S17: TEM characterization of the miniONF nanoagents** 20](#_Toc194070881)

[**Figure S18:** **TEM characterization of the wfONF nanoagents** 21](#_Toc194070882)

[**Figure S19: Development curves of control spheroids**  22](#_Toc194070883)

[**Figure S20: Development curves of spheroids with FasL** 23](#_Toc194070884)

[**Figure S21: Development curves of spheroids with rroOF nanoagent** 24](#_Toc194070885)

[**Figure S22: Development curves of spheroids with miniOF nanoagent** 25](#_Toc194070886)

[**Figure S23: Development curves of spheroids with wfOF nanoagent** 26](#_Toc194070887)

[**Figure S24: Development curves of spheroids with rroONF nanoagent** 27](#_Toc194070888)

[**Figure S25: Development curves of spheroids with miniONF nanoagent** 28](#_Toc194070889)

[**Figure S26: Development curves of spheroids with wfONF nanoagent** 29](#_Toc194070890)

[**Figure S27: Pseudo-phase diagram of cancer spheroid behavior** 30](#_Toc194070891)

[**Figure S28: Population shifts in fluorescence-activated cell sorting (FACS) 31**](#_Toc194070892)

[**Figure S29: FACS data of dissolved spheroids** 32](#_Toc194070893)

[**Figure S30: 2D regrowth of dissolved spheroids** 33](#_Toc194070894)

[**Figure S31: 2D regrowth of dissolved spheroids, titrating concentrations**. 34](#_Toc194070895)

**Table of tables**

[**Table S1: rro DNA origami staples** 36](#_Toc194070896)

[**Table S2: mini DNA origami staples** 41](#_Toc194070897)

[**Table S3: wf DNA origami staples** 42](#_Toc194070898)


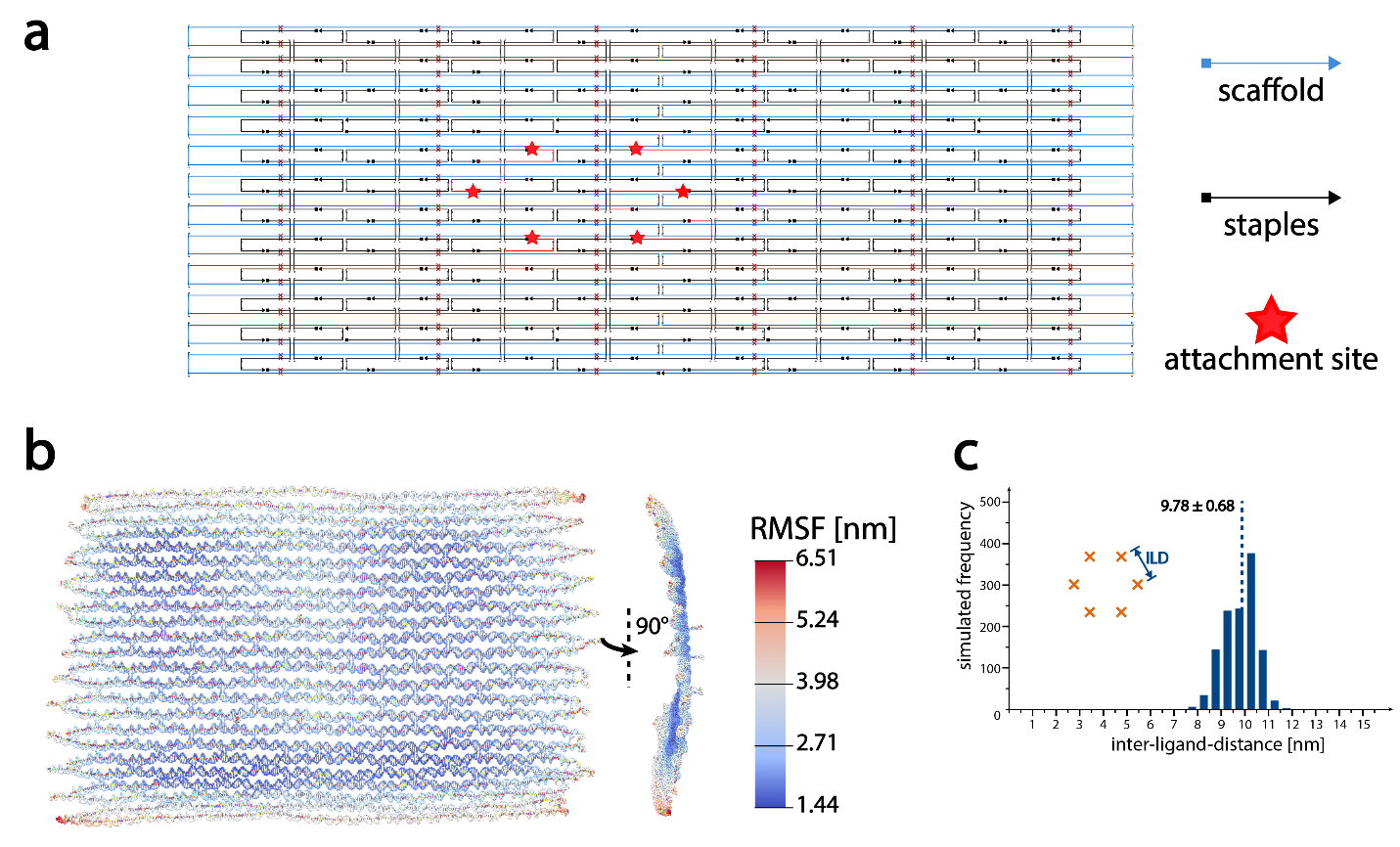


**Figure S1: Design and simulation of the rro DNA origami** (**a**) caDNAno screenshot of the rro layout: blue lines indicate scaffold routing, black lines indicate staple routings and red stars indicate attachment sites. (**b**) oxDNA simulation of the rro: front and side view of the average structure, indicated by a heatmap is the RMSF of the structure. In the oxDNA simulation, the dimensions of the rro DNA origami are approximately 90 nm x 59 nm. (**c**) In-silico analysis of inter-ligand-distances (ILDs) on the respective DNA origami. n=1200 ILDs at different time points in the simulation for each structure. The average ILD extracted from the simulation of the rro origami is 9.78 ± 0.68 nm.


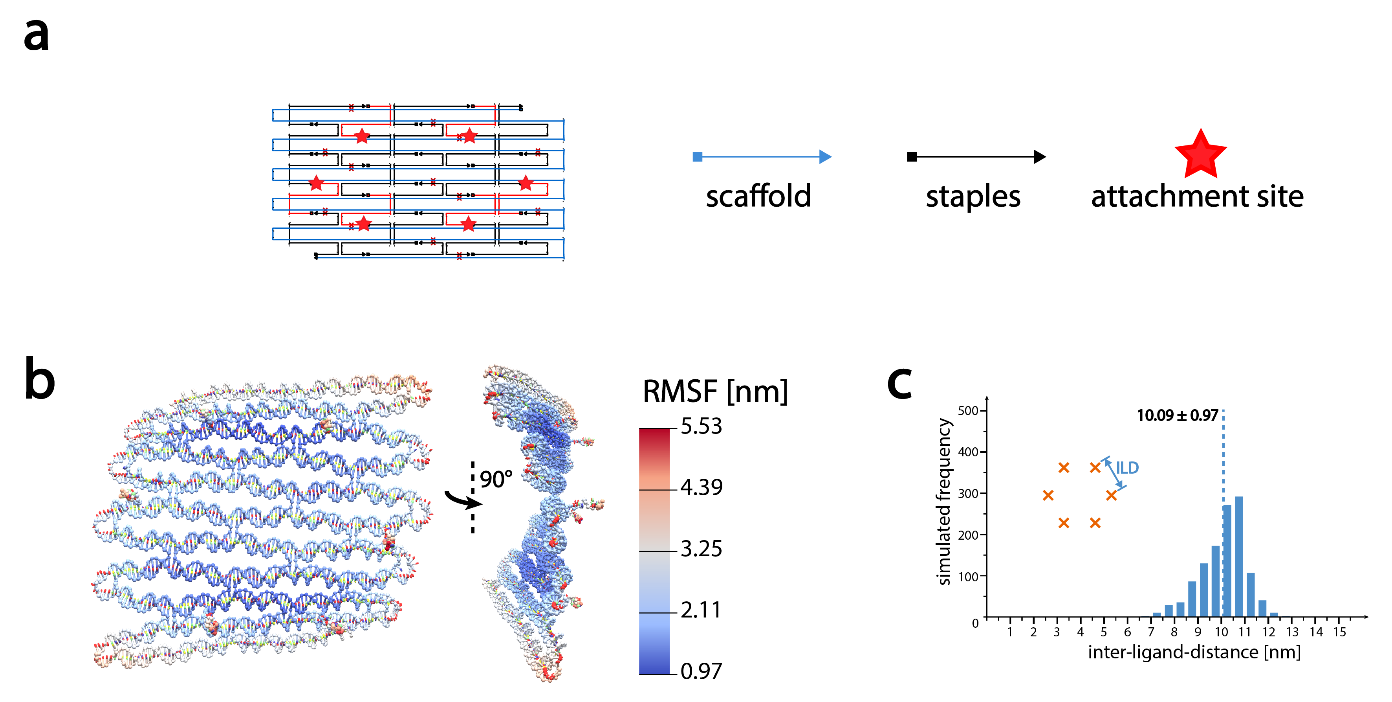


**Figure S2: Design and simulation of the mini DNA** **origami** (**a**) caDNAno screenshot of the mini layout: blue lines indicate scaffold routing, black lines indicate staple routings and red stars indicate attachment sites. (**b**) oxDNA simulation of the mini: front and side view of the average structure, indicated by a heatmap is the RMSF of the structure. In the oxDNA simulation, the dimensions of the mini DNA origami are approximately 28 nm x 24 nm. (**c**) In-silico analysis of ILD on the respective DNA origami. n=1200 ILDs at different time points in the simulation for each structure. The average ILD extracted from the simulation of the mini origami is 10.09 ± 0.97 nm.


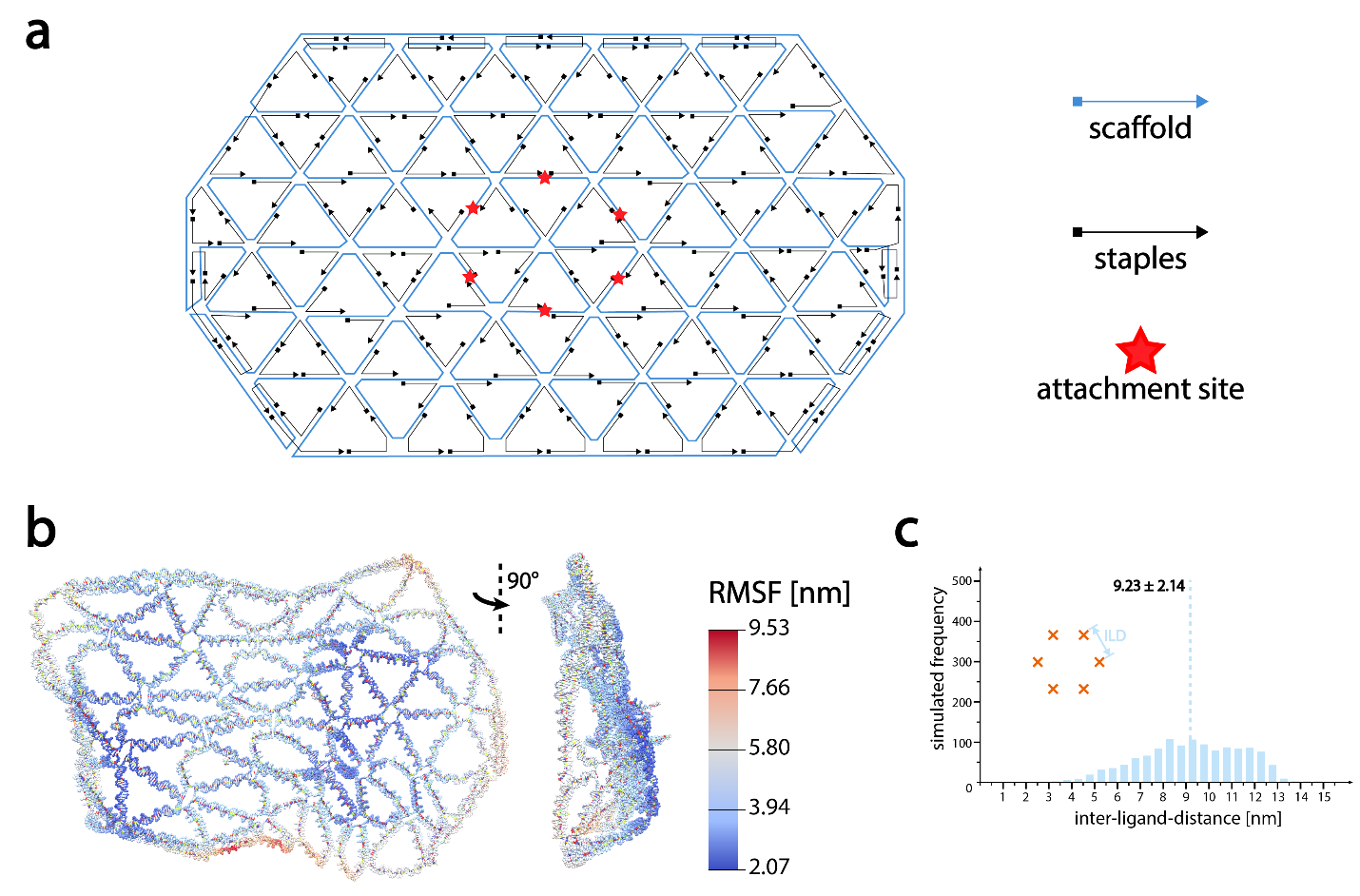


**Figure S3: Design and simulation of the wf DNA origami** (**a**) scaffold and staple layout of the wf layout: blue lines indicate scaffold routing, black lines indicate staple routings and red stars indicate attachment sites. (**b**) oxDNA simulation of the wf: front and side view of the average structure, indicated by a heatmap is the RMSF of the structure. In the oxDNA simulation, the dimensions of the wf DNA origami are approximately 67 nm x 45 nm. (**c**) In-silico analysis of ILD on the respective DNA origami. n=1200 ILDs at different time points in the simulation for each structure. The average ILD extracted from the simulation of the wf origami is 9.23 ± 2.14 nm.


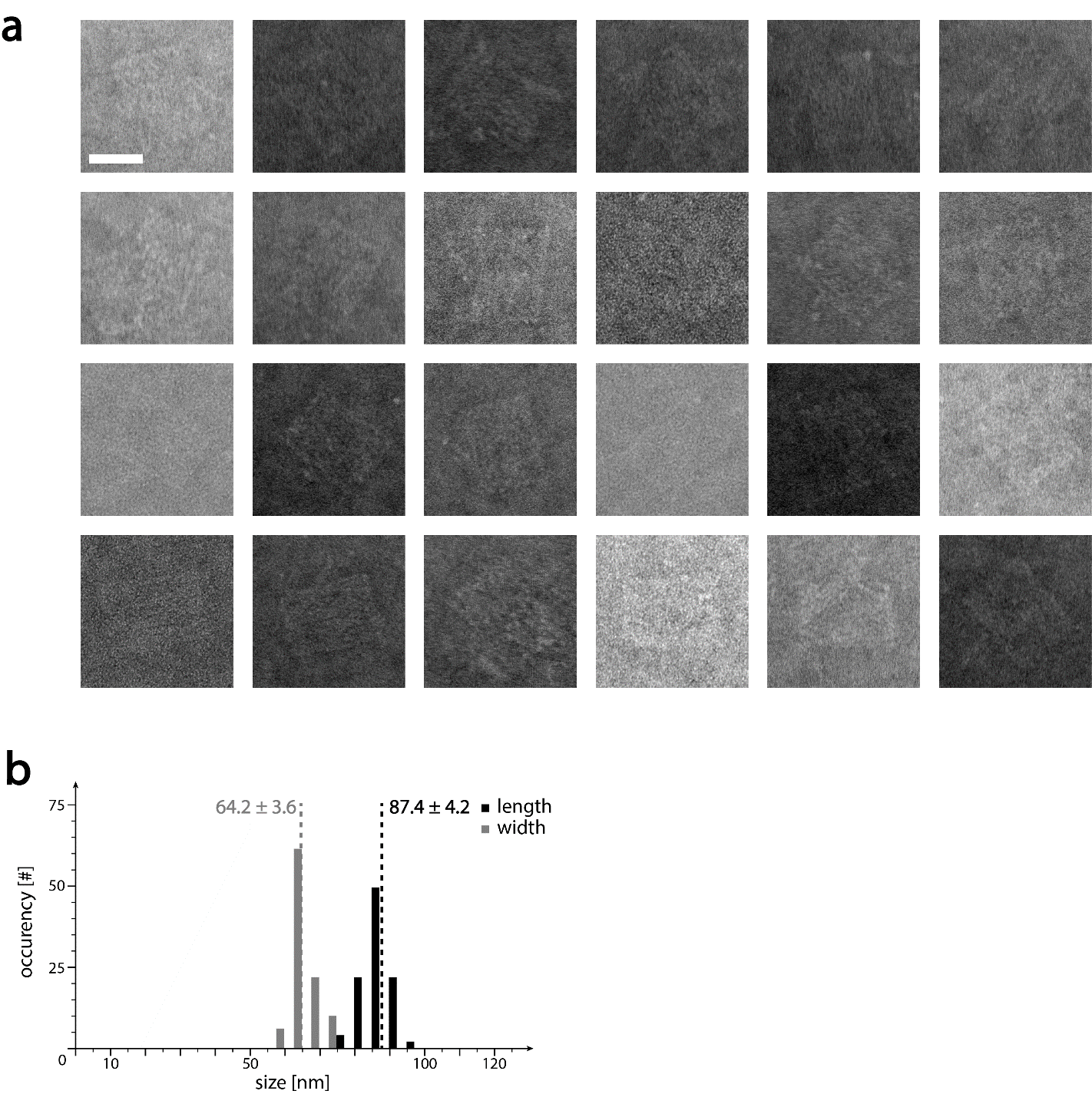


**Figure S4: Transmission electron microscopy (TEM) characterization of the rro DNA origami**: (**a**) Cropped micrographs show single rro origami. (b) Histograms of length (black) and width (grey) of rro origami extracted from TEM micrographs. N>30 structures, normalized to 100. Length is 87.4 ± 4.2 nm and width is 64.2 ± 3.6 nm. Images from the same experiment were used in Figure 1b. The scale bar in (**a**) is 50 nm and holds for all micrographs.


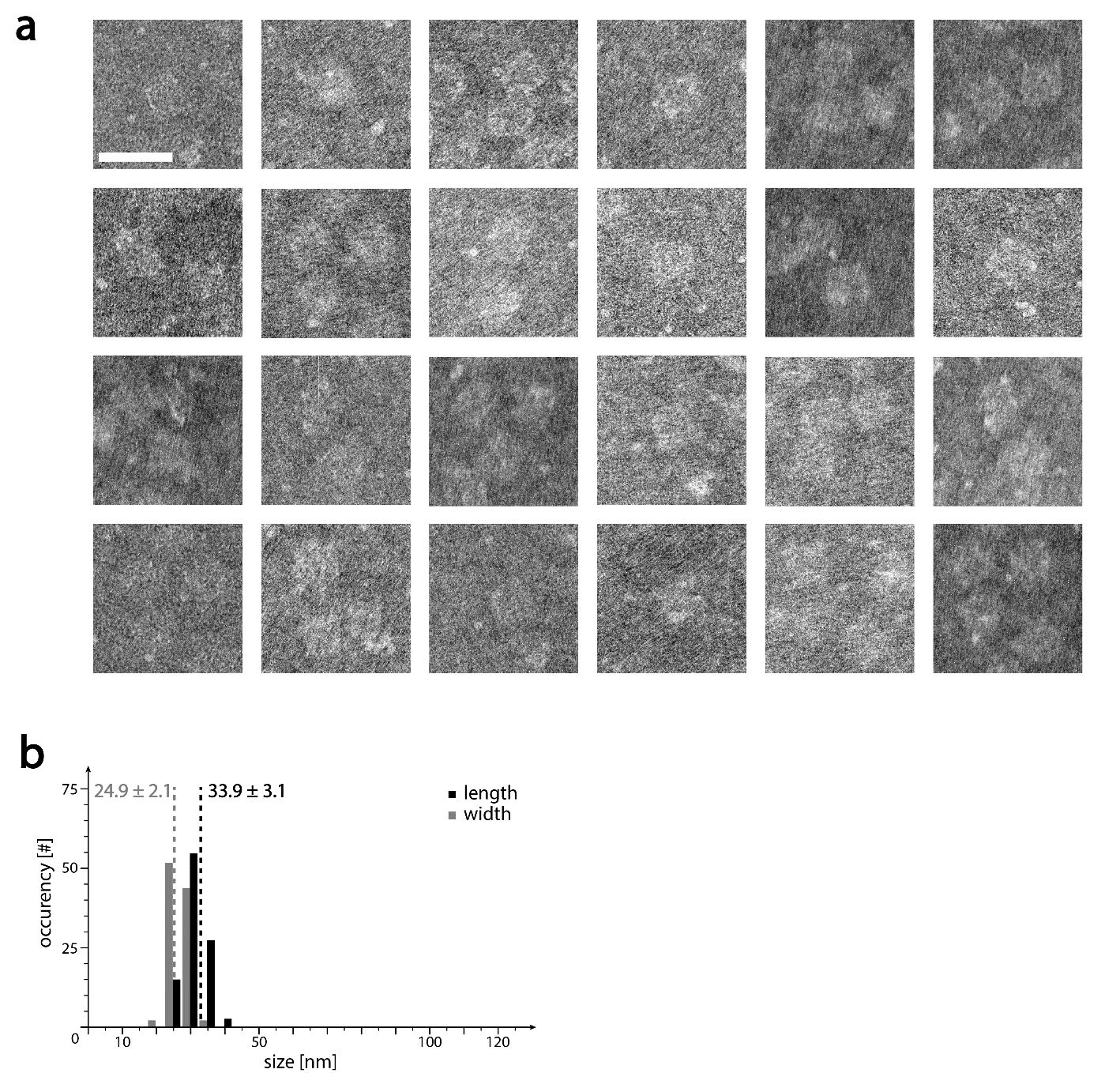


**Figure S5: TEM characterization of the mini DNA origami**: (**a**) Cropped micrographs show mini origami. (b) Histograms of length (black) and width (grey) of mini origami extracted from TEM micrographs. N>30 structures, normalized to 100. Length is 33.9 ± 3.1 nm and width is 24.9 ± 2.1 nm. Images from the same experiment were used in Figure 1b. The scale bar in (**a**) is 50 nm and holds for all micrographs.


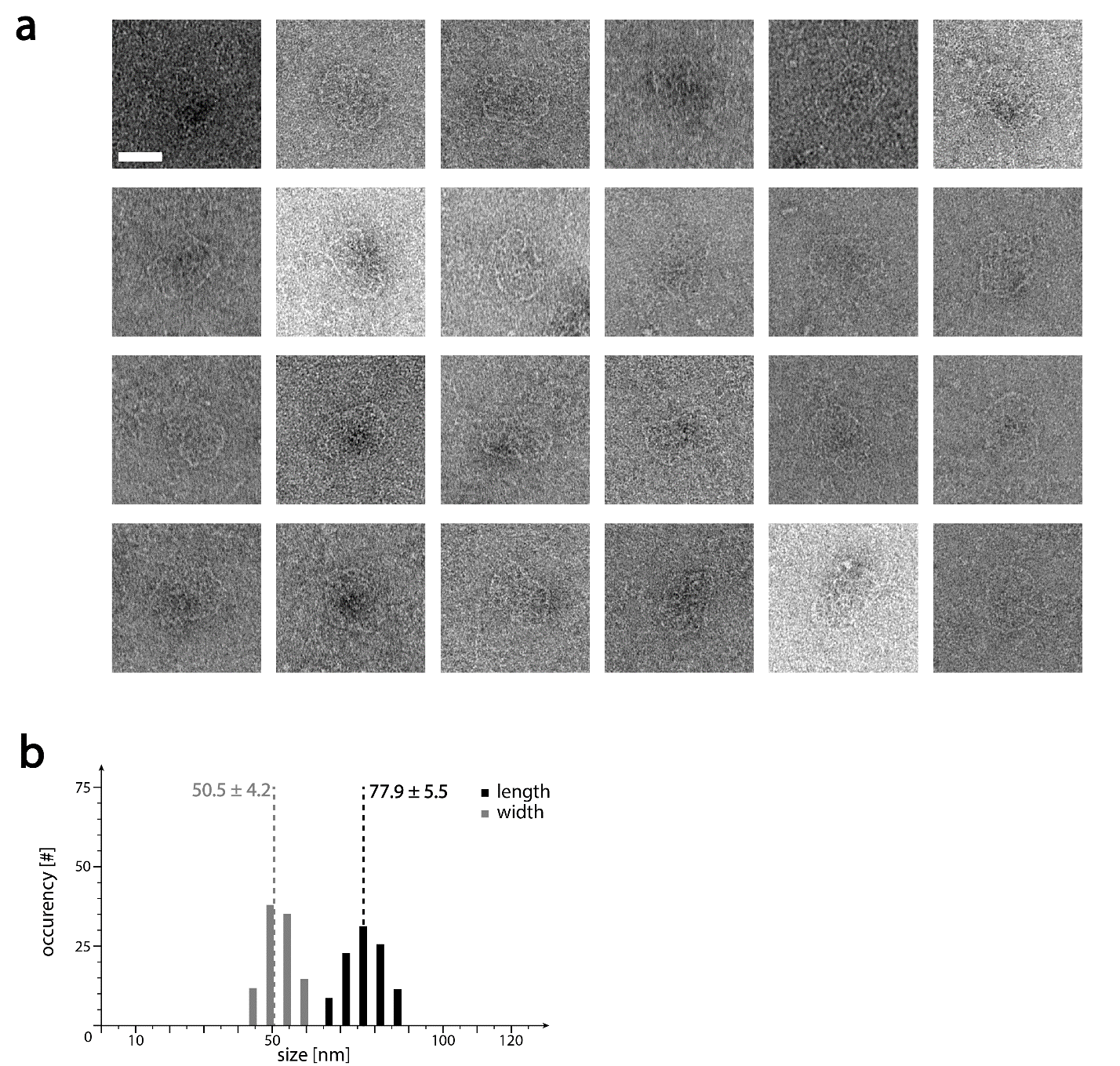


**Figure S6: TEM characterization of the wf DNA origami**: (**a**) Cropped micrographs show single wf origami. (b) Histograms of length (black) and width (grey) of wf origami extracted from TEM micrographs. N>30 structures, normalized to 100. Length is 77.9 ± 5.5 nm and width is 50.5 ± 4.2 nm. Images from the same experiment were used in Figure 1b. The scale bar in (**a**) is 50 nm and holds for all micrographs.


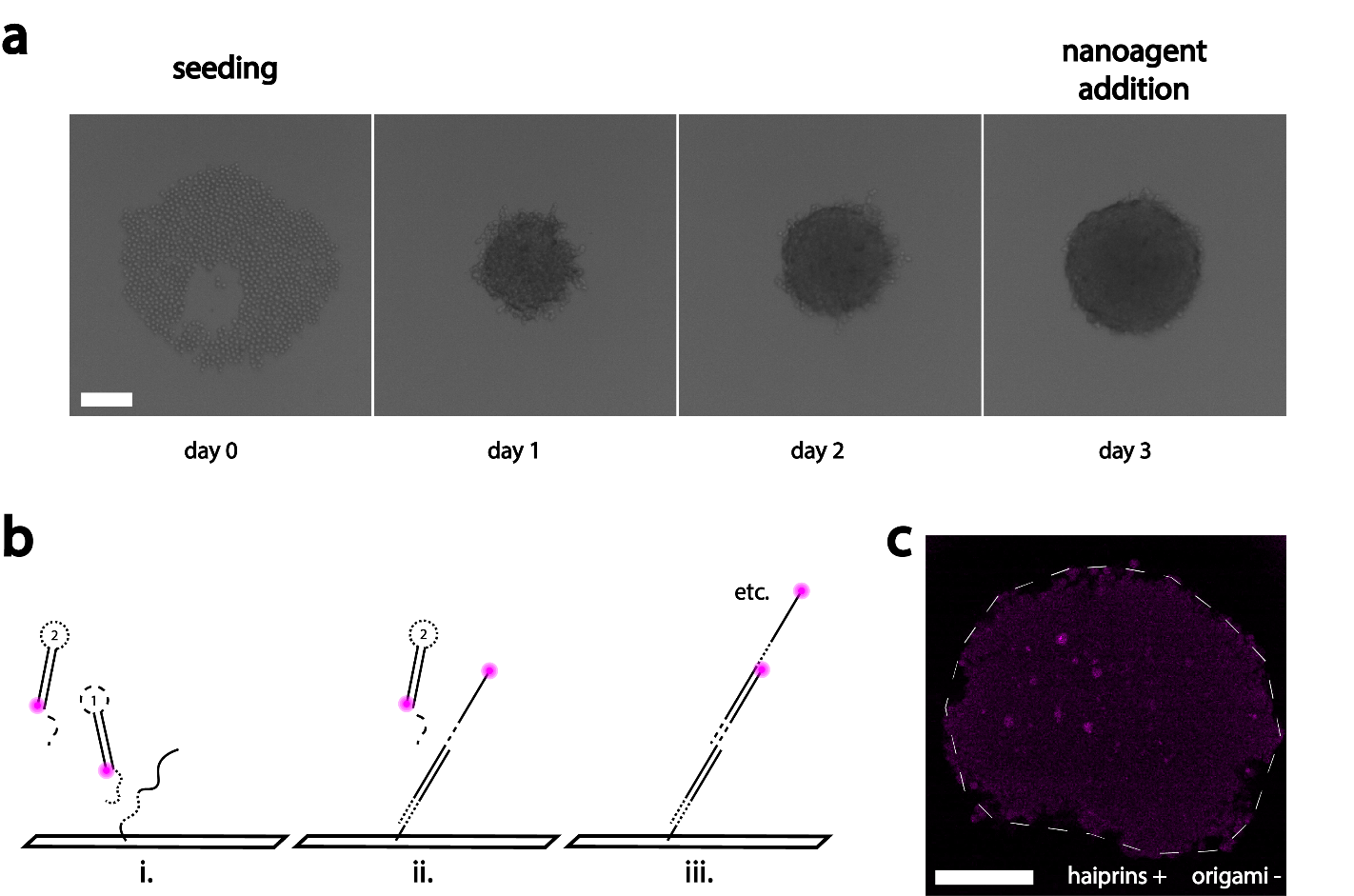


**Figure S7: Cancer spheroid seeding and origami FISH** (**a**) first three days in the spheroid development. On day 0 the cells were seeded and in a flat layer on the low adhesion plate. In the following three days, the cells proliferate and form a spheroid with sharp contours. (**b**) Schematic of origami FISH: staples extended with an anchor sequence are protruding from a DNA origami. (i.) To a toehold region (dotted part) on the anchor sequence, hairpin 1 hybridizes and displaces the stem-loop, making a toehold (stripes) and an attachment site for hairpin 2 accessible. (ii.) Displacement of the stem-loop in hairpin 2, again makes a toehold and an attachment site for hairpin 1 accessible, starting a chain reaction, which leads to (iii.) an accumulation of fluorophores around the initial attachment site. (**c**) Control spheroid without DNA origami, but with fluorescent hairpins shows a very dim, homogeneous hairpin/FISH fluorescence signal throughout the entire spheroid. The contrast was adjusted for better visibility. The scale bar in (**a**) is 100 µm and in (**c**) is 200 µm.


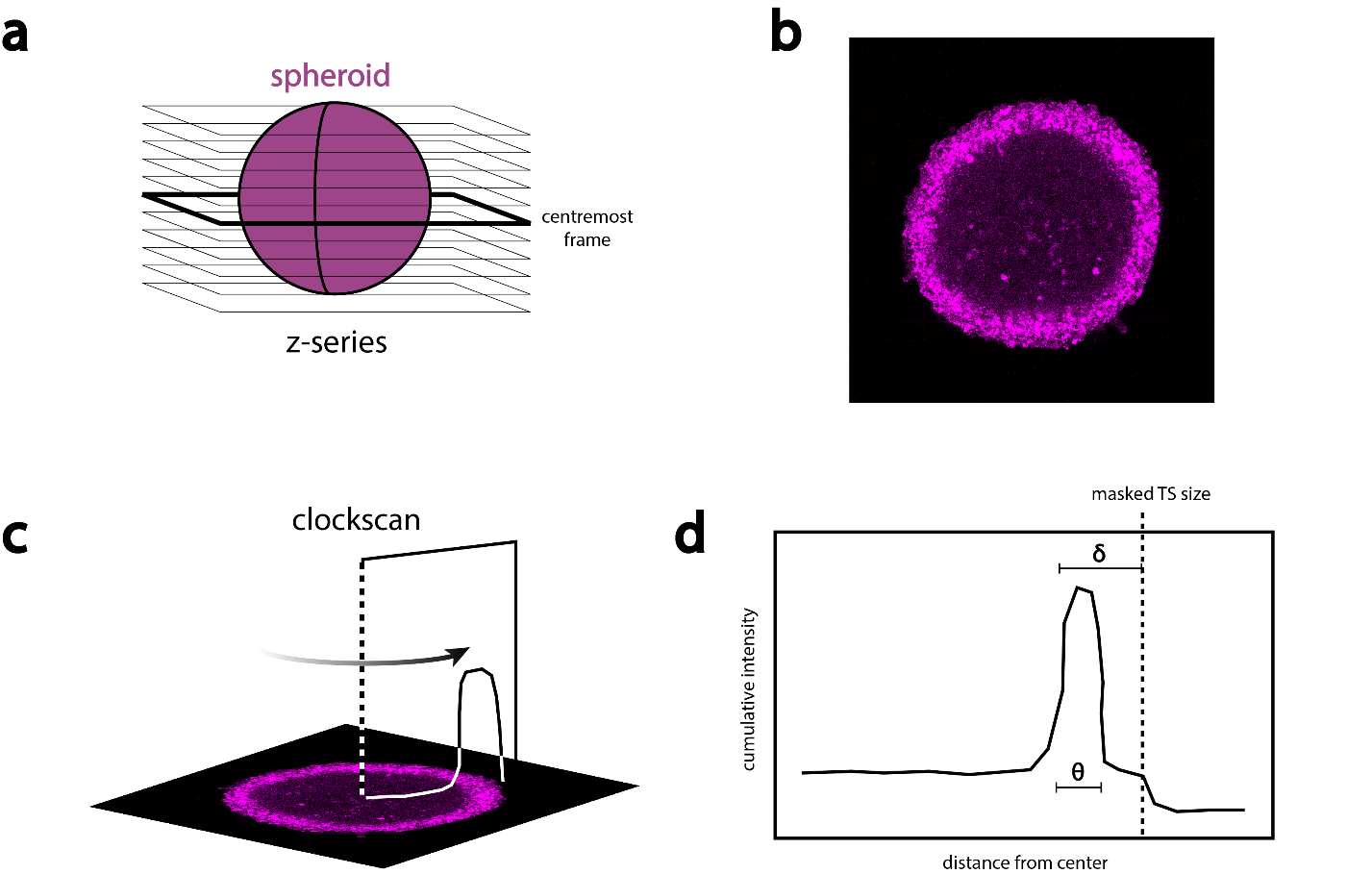


**Figure S8: Schematic of the clockscan protocol** used to extract the penetration depth from fluorescence images. (**a**) A z-stack of a cleared spheroid was recorded with a confocal microscope and the thickest part on the centermost frame (**b**) was determined. On the thickest part of the spheroid a clockscan (**c**) was performed, averaging the fluorescence signal from the center towards the outside. The fluorescence intensity was then plotted (**d**) and the penetration depth and the ring thickness were extracted.


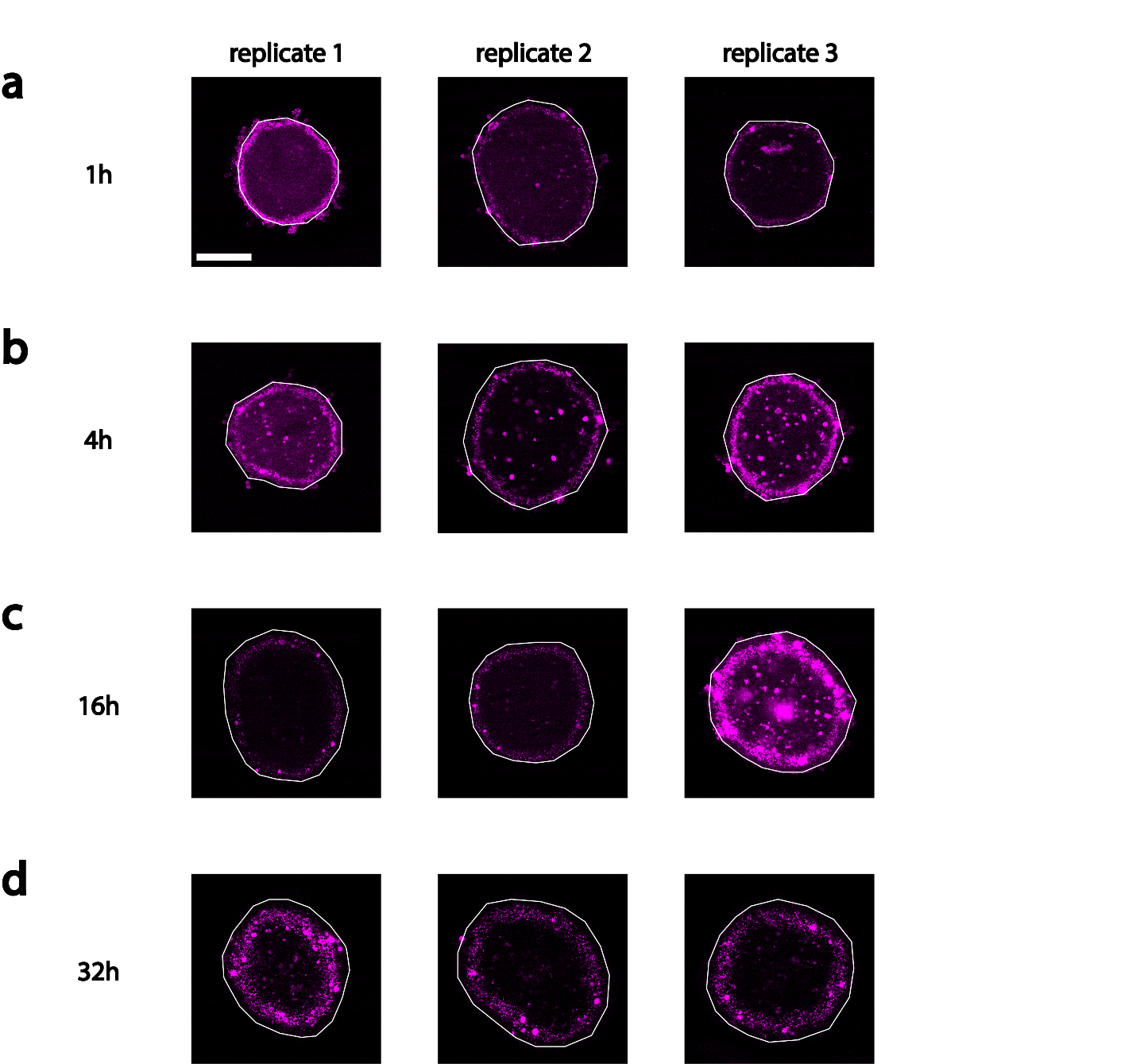


**Figure S9: rro origami penetration through cancer spheroids** after (**a**) 1 hour, (**b**) 4 hours, (**c**) 16 hours, and (**d**) 32 hours, as triplicates of the same condition. The actual spheroid outlines, extracted from the GFP channel, are indicated as white lines. Graphs in Figure 2e are based on these images, and one 16h image was also used in Figure 2d. The contrast was adjusted in some images for better visibility of the fluorescent rings. The scale bar is 200 µm and holds for all images.


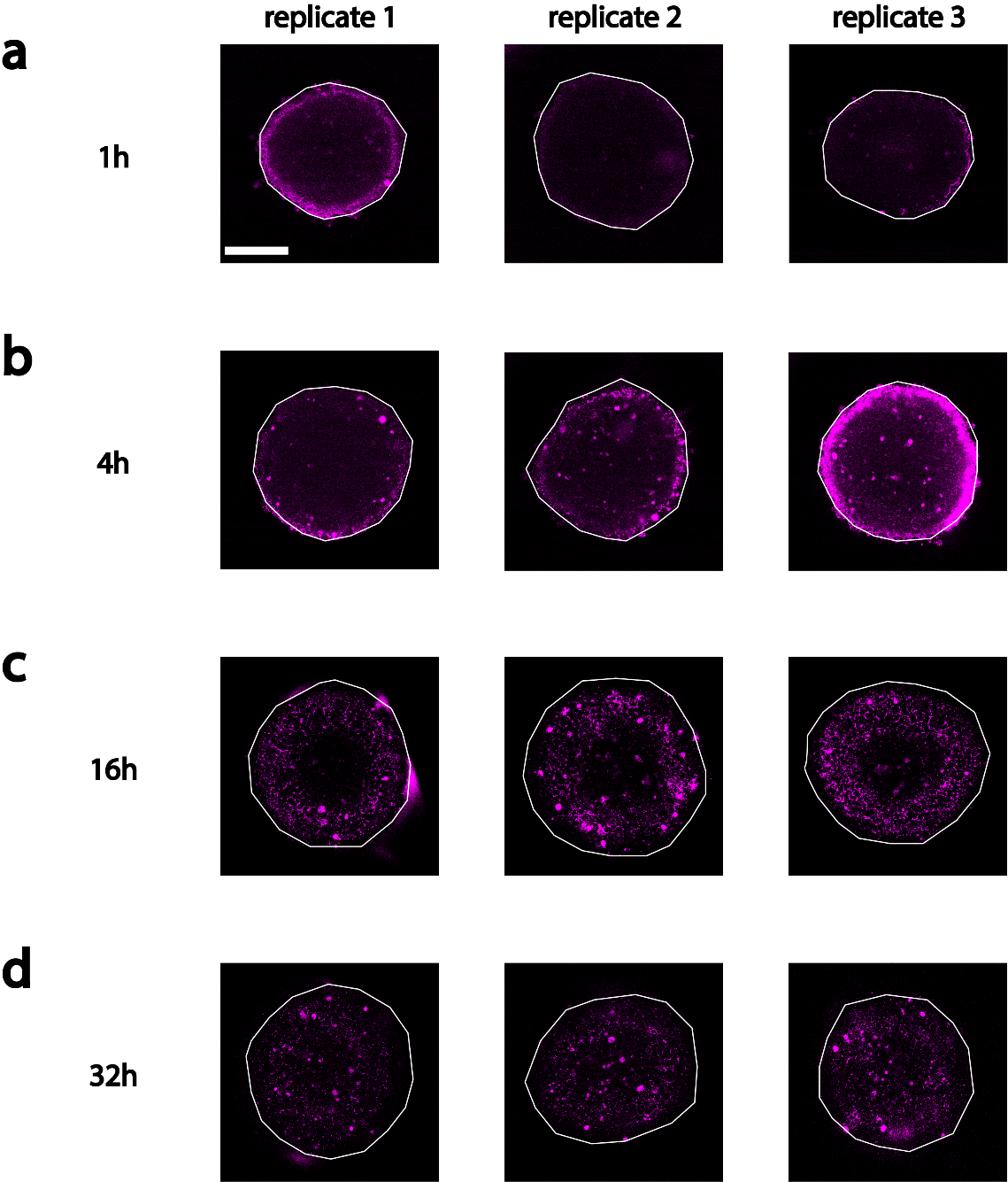


**Figure S10: mini origami penetration through spheroids** after (**a**) 1 hour, (**b**) 4 hours, (**c**) 16 hours, and (**d**) 32 hours, as triplicates of the same condition. The actual spheroid outlines, extracted from the GFP channel, are indicated as white lines. Graphs in Figure 2e are based on these images, and one 16h image was also used in Figure 2d. The contrast was adjusted in some images for better visibility of the fluorescent rings. The scale bar is 200 µm and holds for all images.


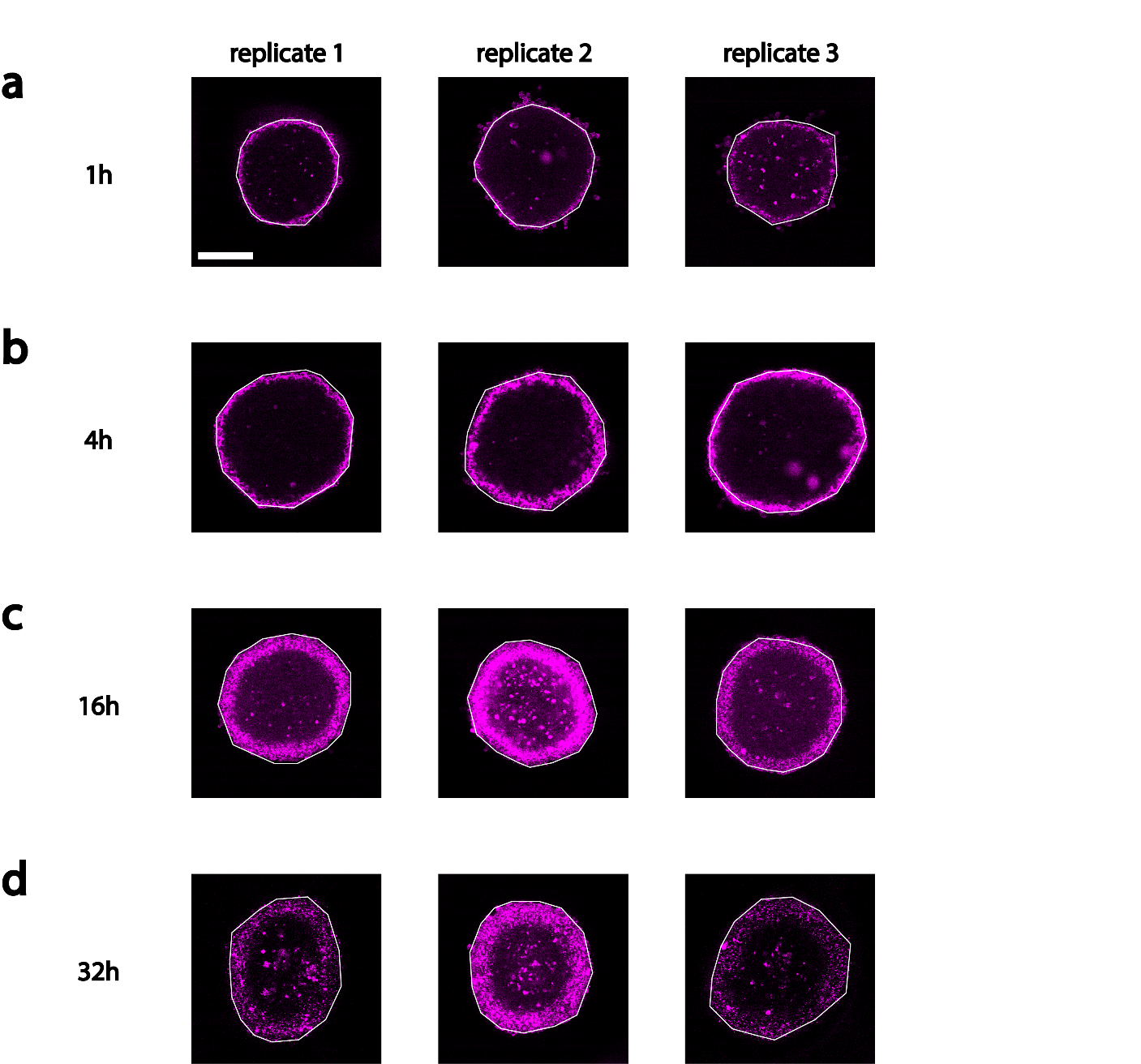


**Figure S11: wf origami penetration through spheroids** after (**a**) 1 hour, (**b**) 4 hours, (**c**) 16 hours, and (**d**) 32 hours, as triplicates of the same condition. The actual spheroid outlines, extracted from the GFP channel, are indicated as white lines. Graphs in Figure 2e are based on these images, and one 16h image was also used in Figure 2d. The contrast was adjusted in some images for better visibility of the fluorescent rings. The scale bar is 200 µm and holds for all images.

**
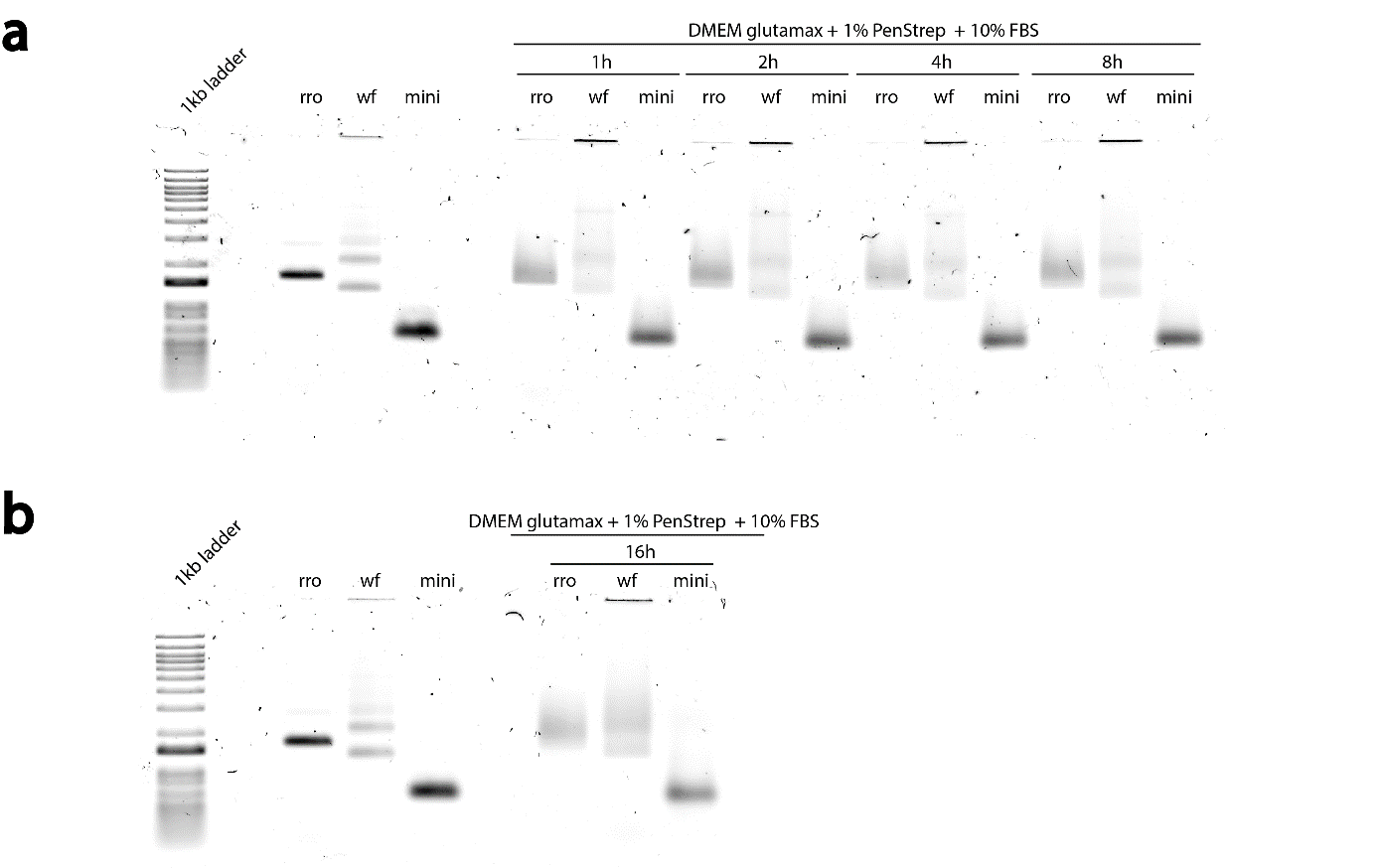
**

**Figure S12: Stability test of DNA origami in serum-containing medium using agarose gel electrophoresis.** The three types of DNA origami (rro, wf, mini) were exposed to cell culture medium (Dulbecco's modified eagle medium (DMEM) gluatamax, supplemented with penicillin/ streptomycin (PenStrep) to 1 % and fetal bovine serum (FBS) to 10 %) for (**a**) 1 to 8 hours and (**b**) 16 hours at 37 °C. The DNA origami migrate slower with increasing structure size. Incubation in cell culture medium led to an increase in smear and, in case of wf origami an increase of aggregates. 200 ng of each DNA origami were used, respectively.


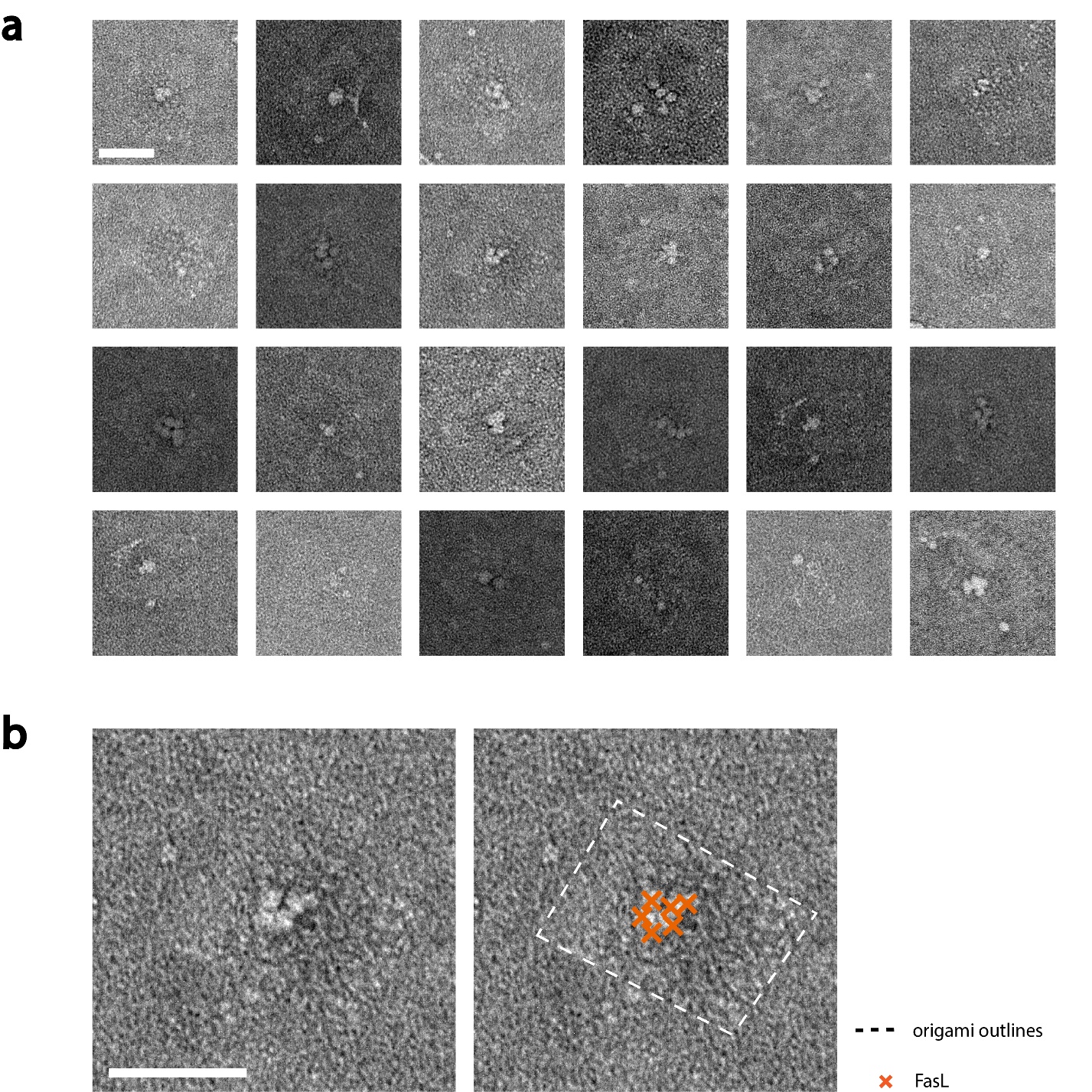


**Figure S13: TEM characterization of the rroOF nanoagents**. (**a**) Cropped micrographs show single rro DNA origami with FasL attached to them. FasL proteins are identifiable as white spots. (**b**) Zoom-in of one rroOF nanoagent, duplicate on the right with DNA origami outlines and proteins marked. The attachment efficiency of FasL to the nanoagent was determined to be 71 % in a previous publication[1]. The scale bars are 50 nm and hold for all micrographs of the respective subfigure.


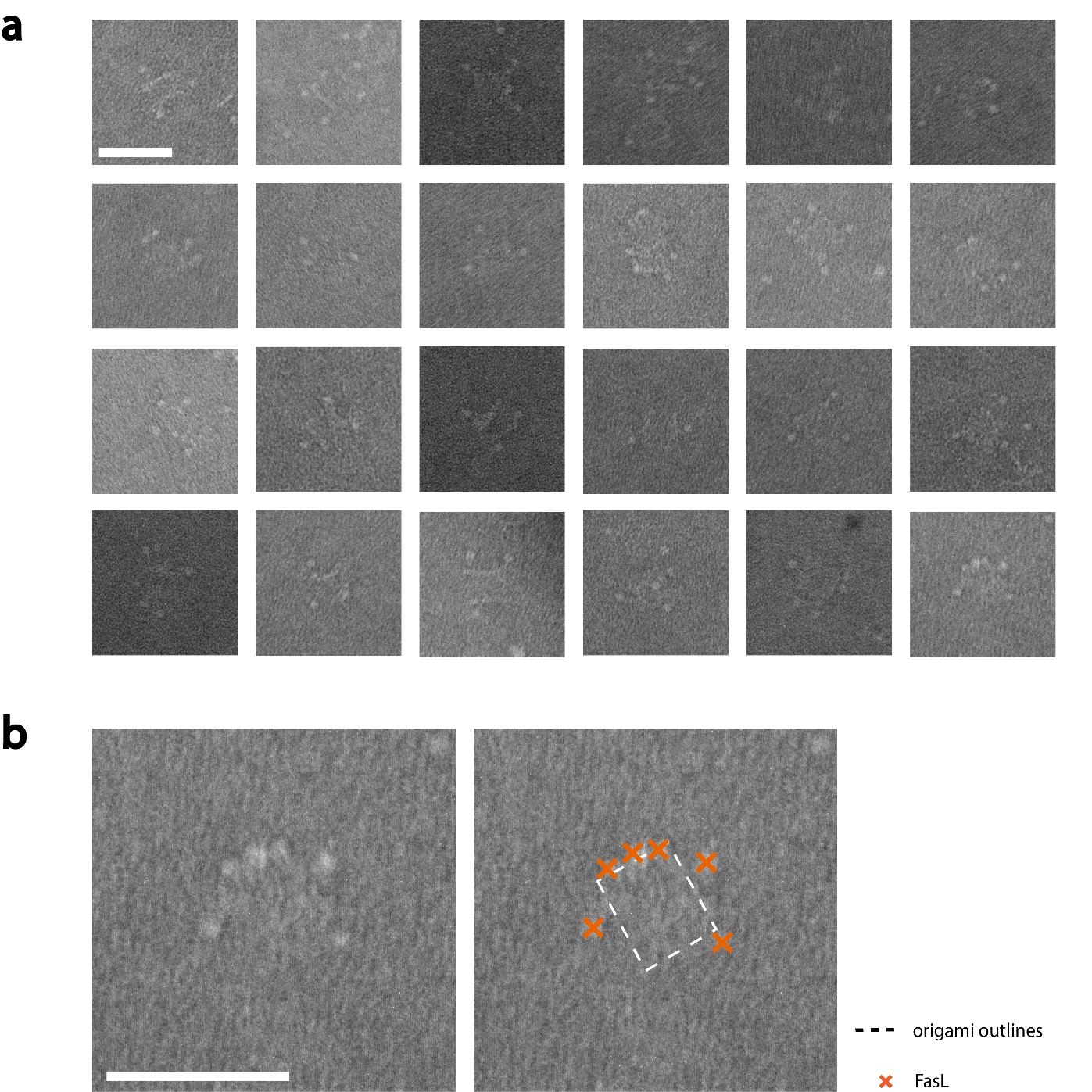


**Figure S14: TEM characterization of the miniOF nanoagents**. (**a**) Cropped micrographs show single mini DNA origami with FasL attached to them. FasL proteins are identifiable as white spots. (**b**) Zoom-in of one miniOF nanoagent, duplicate on the right with DNA origami outlines and proteins marked. The attachment efficiency of FasL to the nanoagent was determined to be 71 % in a previous publication[1]. The scale bars are 50 nm and hold for all micrographs of the respective subfigure.


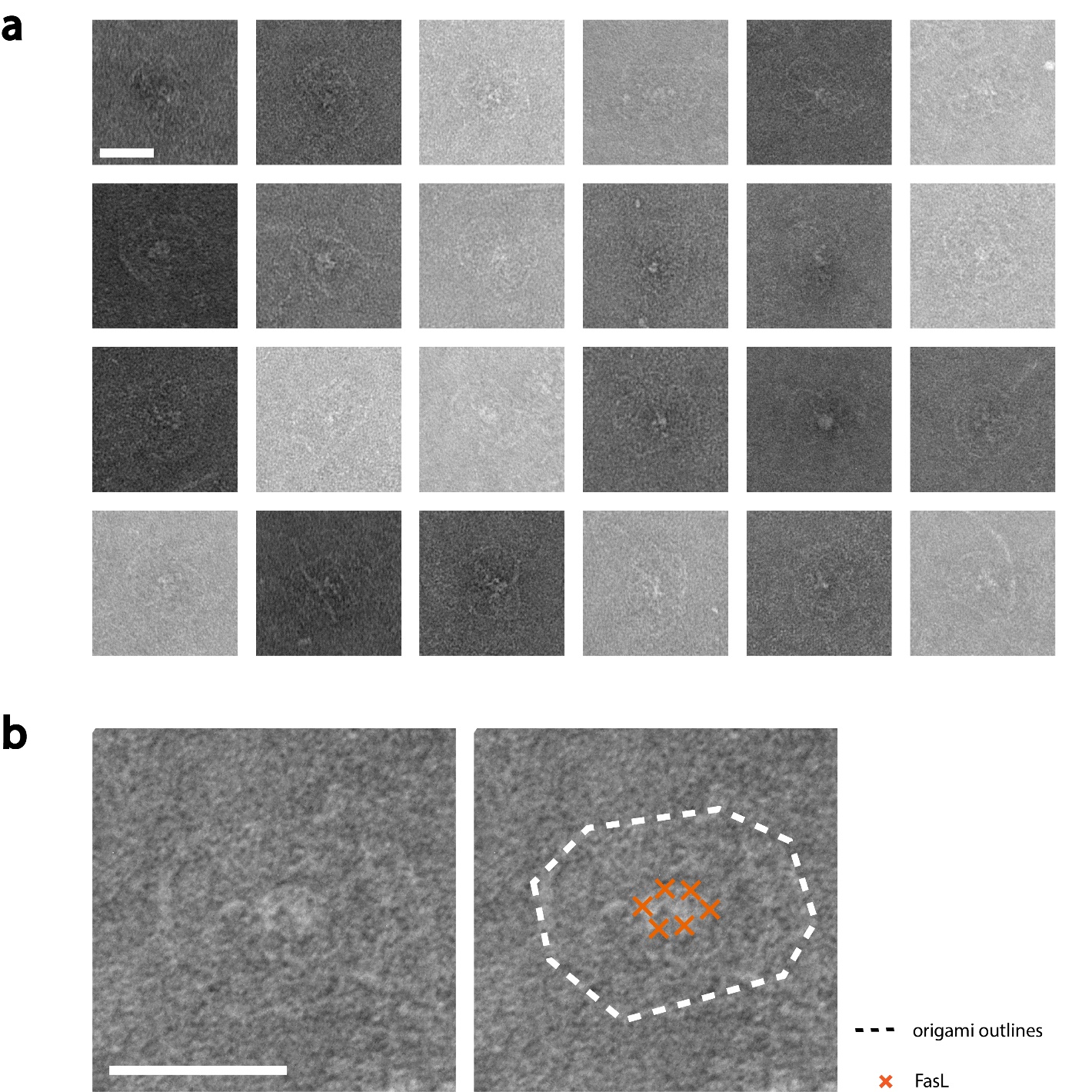


**Figure S15: TEM characterization of the wfOF nanoagents:** (**a**) Cropped micrographs show single wf DNA origami with FasL attached to them. FasL proteins are identifiable as white spots. (**b**) Zoom-in of one wfOF nanoagent, duplicate on the right with DNA origami outlines and proteins marked. The attachment efficiency of FasL to the nanoagent was determined to be 71 % in a previous publication[1]. The scale bars are 50 nm and hold for all micrographs of the respective subfigure.


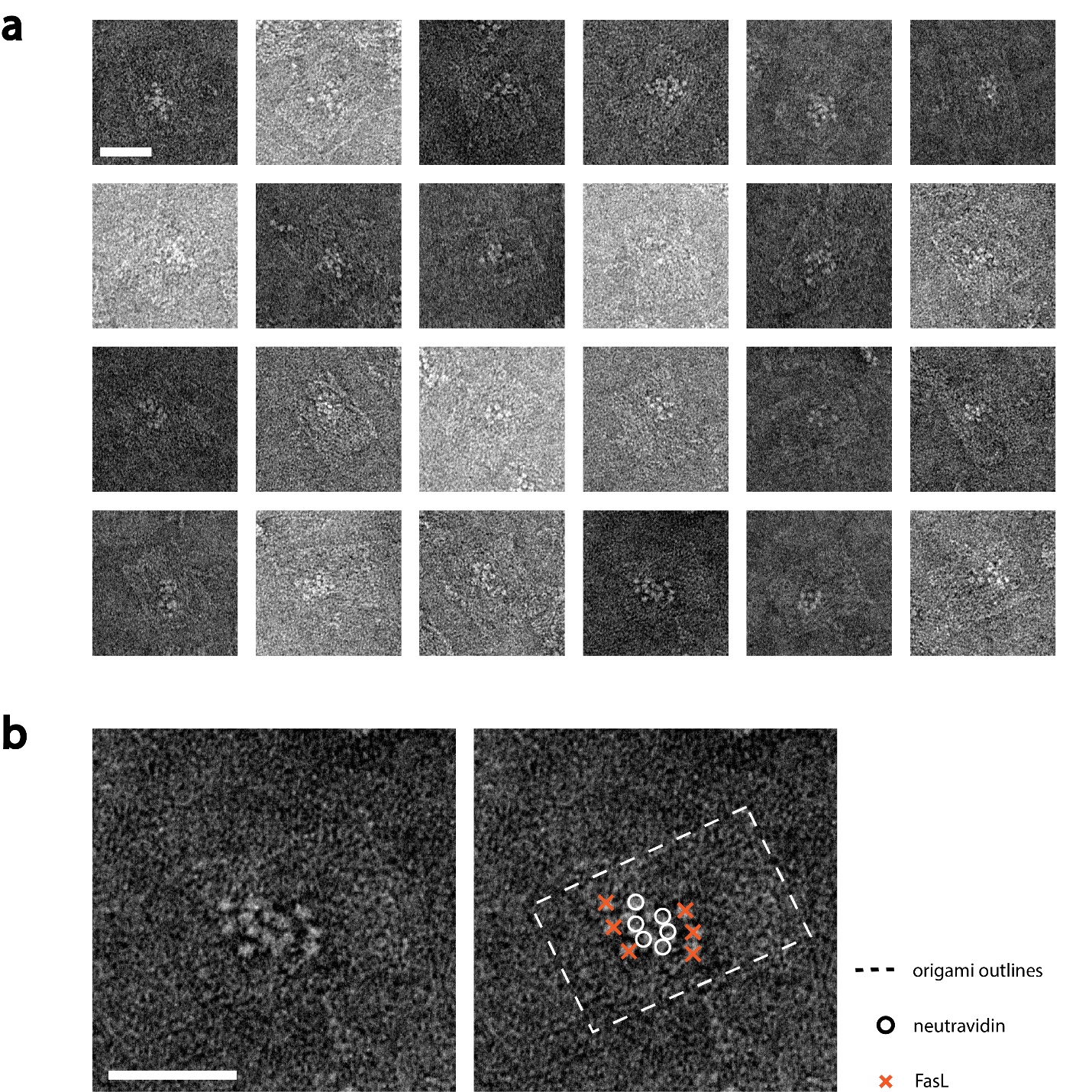


**Figure S16: TEM characterization of the rroONF nanoagents:** (**a**) Cropped micrographs show single rro DNA origami with neutravidin and FasL attached to them. Neutravidin and FasL proteins are identifiable as white spots. (**b**) Zoom-in of one rroONF nanoagent, duplicate on the right with DNA origami outlines and proteins marked. The attachment efficiency of FasL to the nanoagent was determined to be 76 % in a previous publication[2]. The scale bars are 50 nm and hold for all micrographs of the respective subfigure.


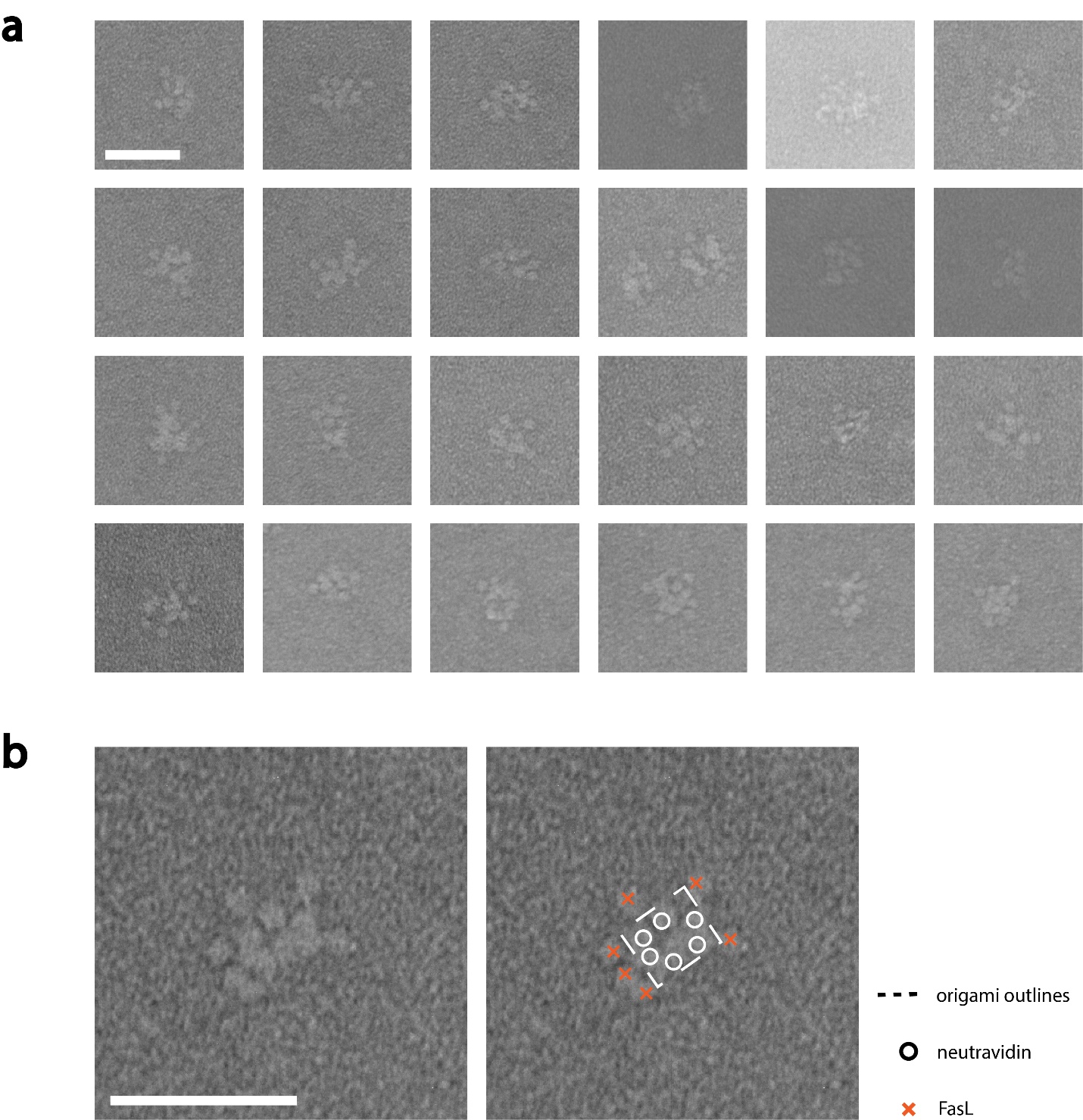


**Figure S17: TEM characterization of the miniONF nanoagents:** (**a**) Cropped micrographs show single mini DNA origami with neutravidin and FasL attached to them. Neutravidin and FasL proteins are identifiable as white spots. (**b**) Zoom-in of one miniONF nanoagent, duplicate on the right with DNA origami outlines and proteins marked. The attachment efficiency of FasL to the nanoagent was determined to be 76 % in a previous publication[2]. The scale bars are 50 nm and hold for all micrographs of the respective subfigure.


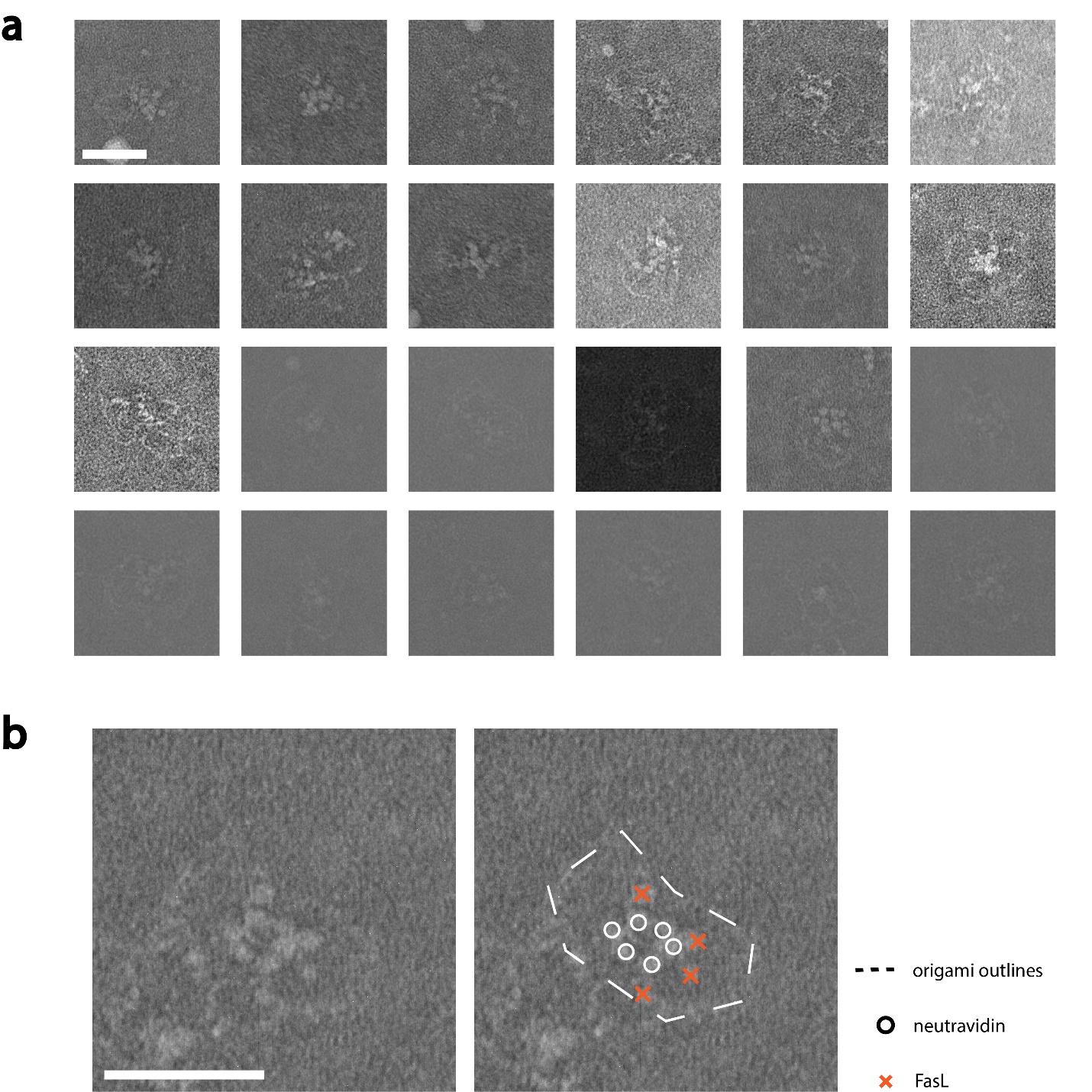


**Figure S18:** **TEM characterization of the wfONF nanoagents:** (**a**) Cropped micrographs show single wf DNA origami with neutravidin and FasL attached to them. Neutravidin and FasL proteins are identifiable as white spots. (**b**) Zoom-in of one wfONF nanoagent, duplicate on the right with DNA origami outlines and proteins marked. The attachment efficiency of FasL to the nanoagent was determined to be 76 % in a previous publication[2]. The scale bars are 50 nm and hold for all micrographs of the respective subfigure.


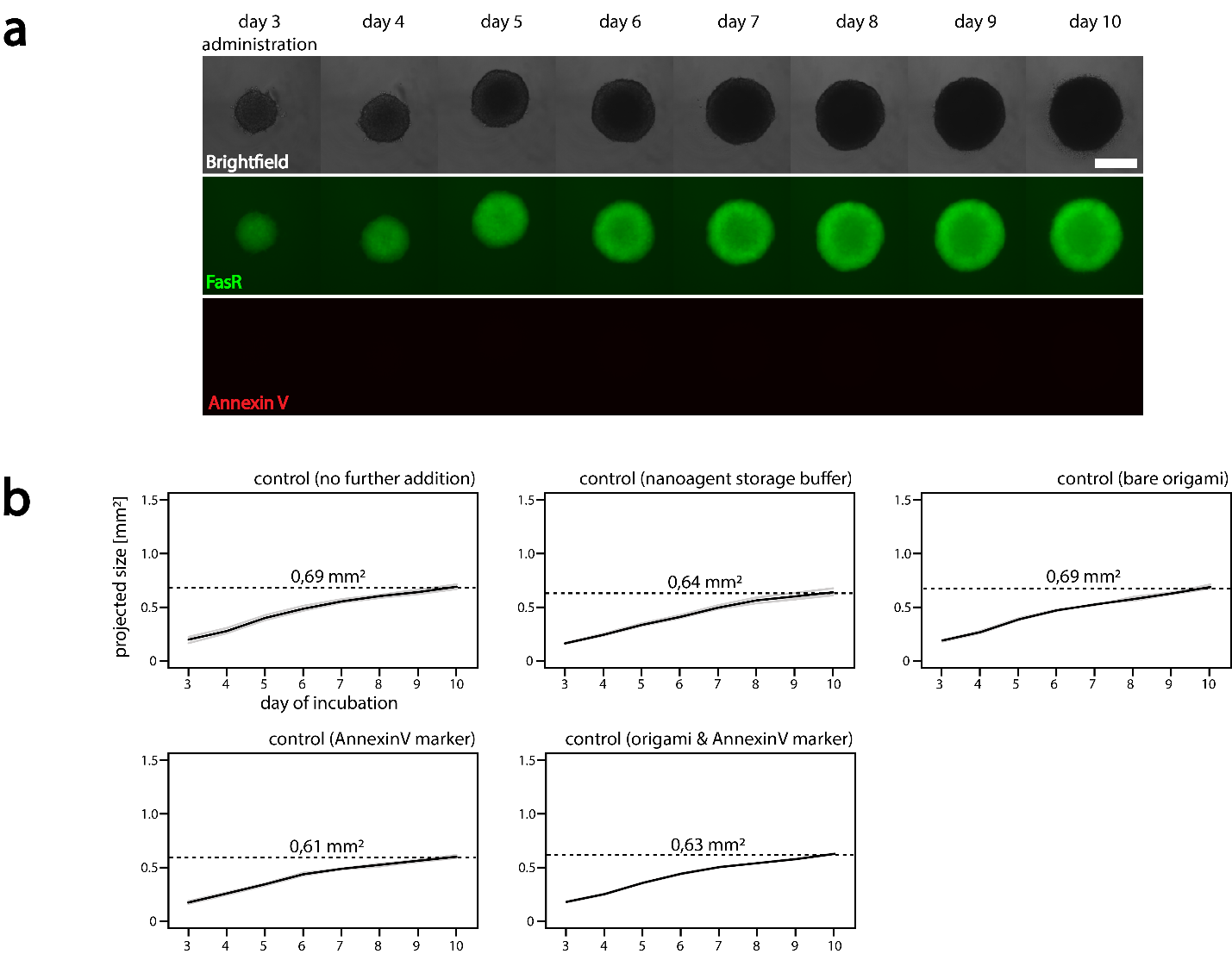


**Figure S19: Development curves of control spheroids** (**a**) Brightfield, GFP (FasR), and Texas Red (Annexin V) images of a spheroid over 7 d (from day 3 to day 10). (**b**) Size projections of spheroids incubated with no additives, nanoagent storage buffer, DNA origami, AnnexinV marker, and the combination of Annexin V marker and DNA origami. Data were also used in Figure 3. The scale bar in (**a**) is 500 µm and holds for all images. Thin grey lines indicate single experiments and thick black lines indicate averages of those (n=3).


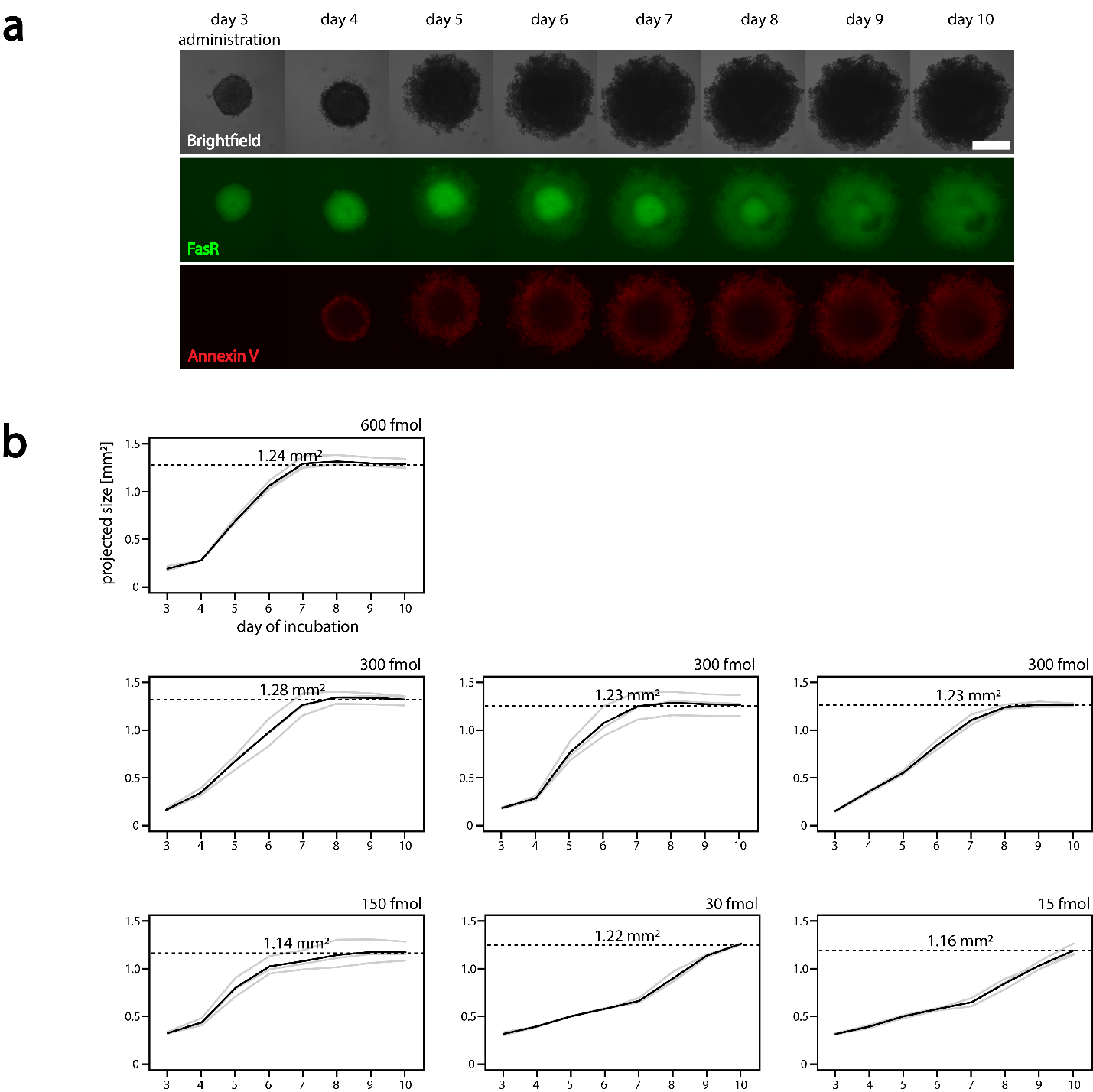


**Figure S20: Development curves of spheroids with FasL** (**a**) Brightfield, GFP (FasR), and Texas Red (Annexin V) images of a spheroid incubated with 300 fmol of FasL over 7 d (from day 3 to day 10). (**b**) Size projections of spheroids incubated with 600 fmol, 300 fmol (triplicate of triplicates), 150 fmol, 30 fmol, and 15 fmol nanoagent. Data were also used in Figure 3. The scale bar in (**a**) is 500 µm and holds for all images. Thin grey lines indicate single experiments and thick black lines indicate averages of those (n=3).


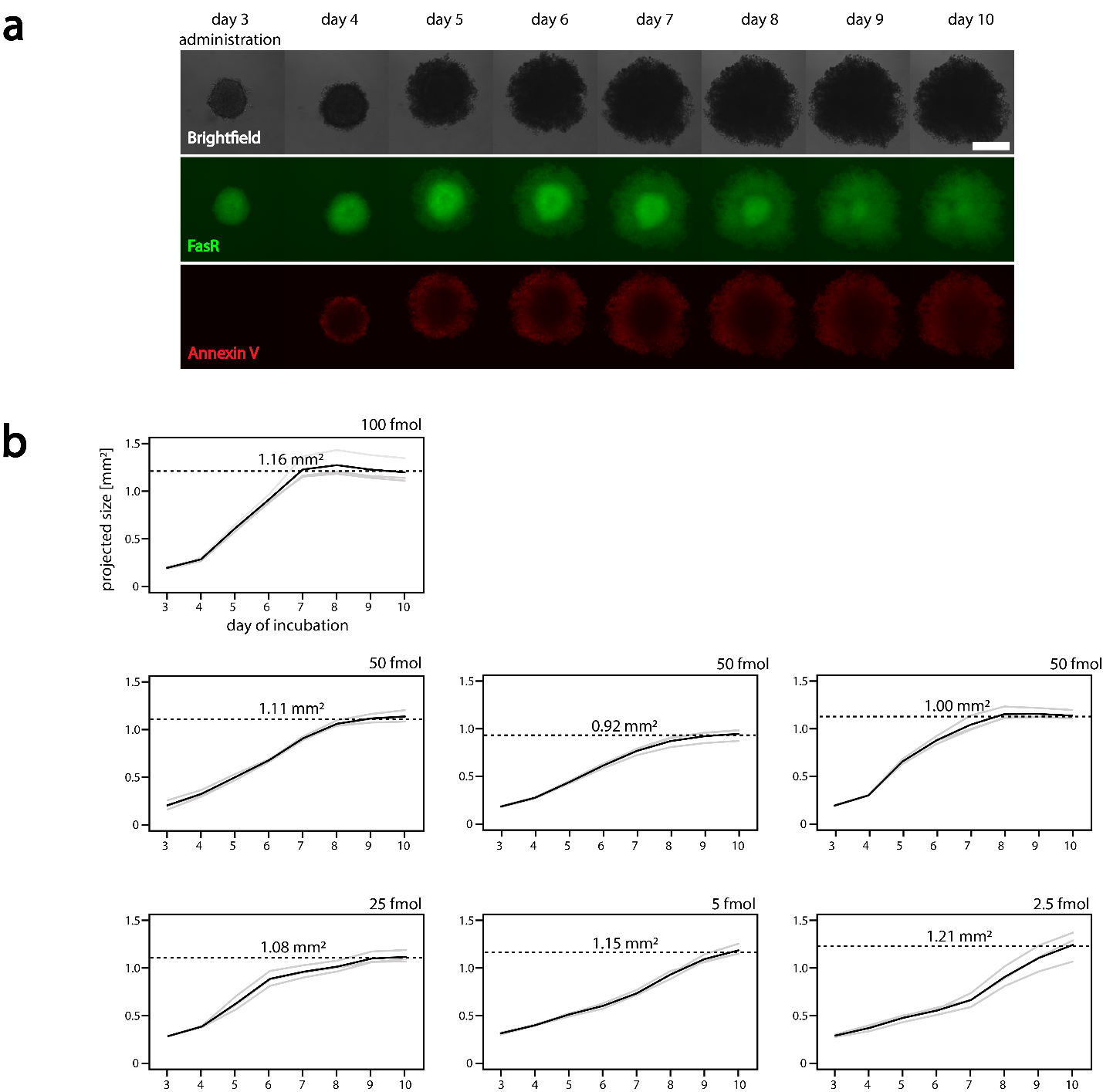


**Figure S21: Development curves of spheroids with rroOF nanoagent** (**a**) Brightfield, GFP (FasR), and Texas Red (Annexin V) images of a spheroid with 50 fmol of rroOF nanoagent over 7 d (from day 3 to day 10). (**b**) Size projections of spheroids incubated with 100 fmol, 50 fmol (triplicate of triplicates), 25 fmol, 5 fmol, and 2.5 fmol nanoagent. Data were also used in Figure 3. The scale bar in (**a**) is 500 µm and holds for all images. Thin grey lines indicate single experiments and thick black lines indicate averages of those (n=3).


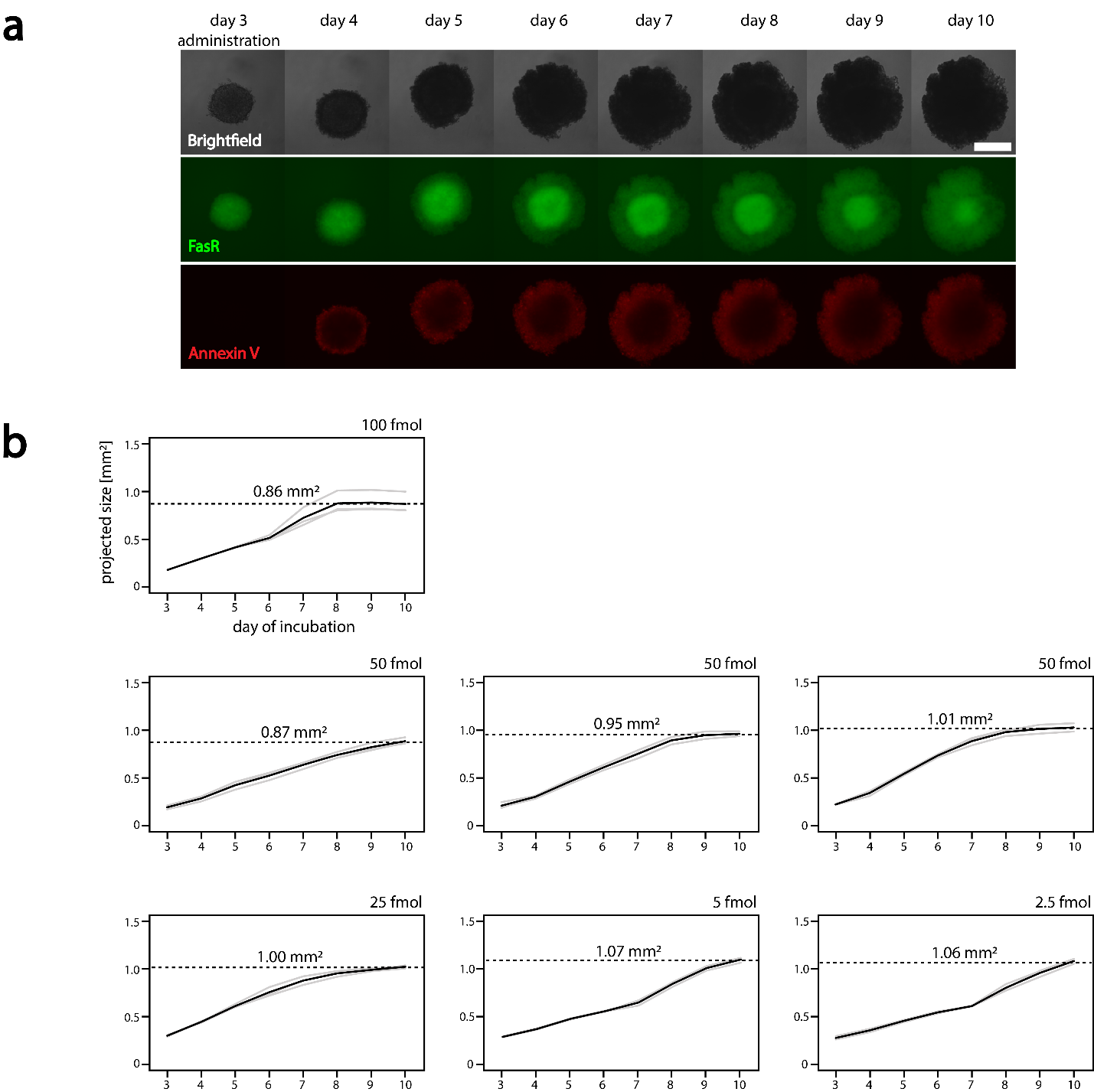


**Figure S22: Development curves of spheroids with miniOF nanoagent** (**a**) Brightfield, GFP (FasR), and Texas Red (Annexin V) images of a spheroid with 50 fmol of miniOF nanoagent over 7 d (from day 3 to day 10). (**b**) Size projections of spheroids incubated with 100 fmol, 50 fmol (triplicate of triplicates), 25 fmol, 5 fmol, and 2.5 fmol nanoagent. Data were also used in Figure 3. The scale bar in (**a**) is 500 µm and holds for all images. Thin grey lines indicate single experiments and thick black lines indicate averages of those (n=3).


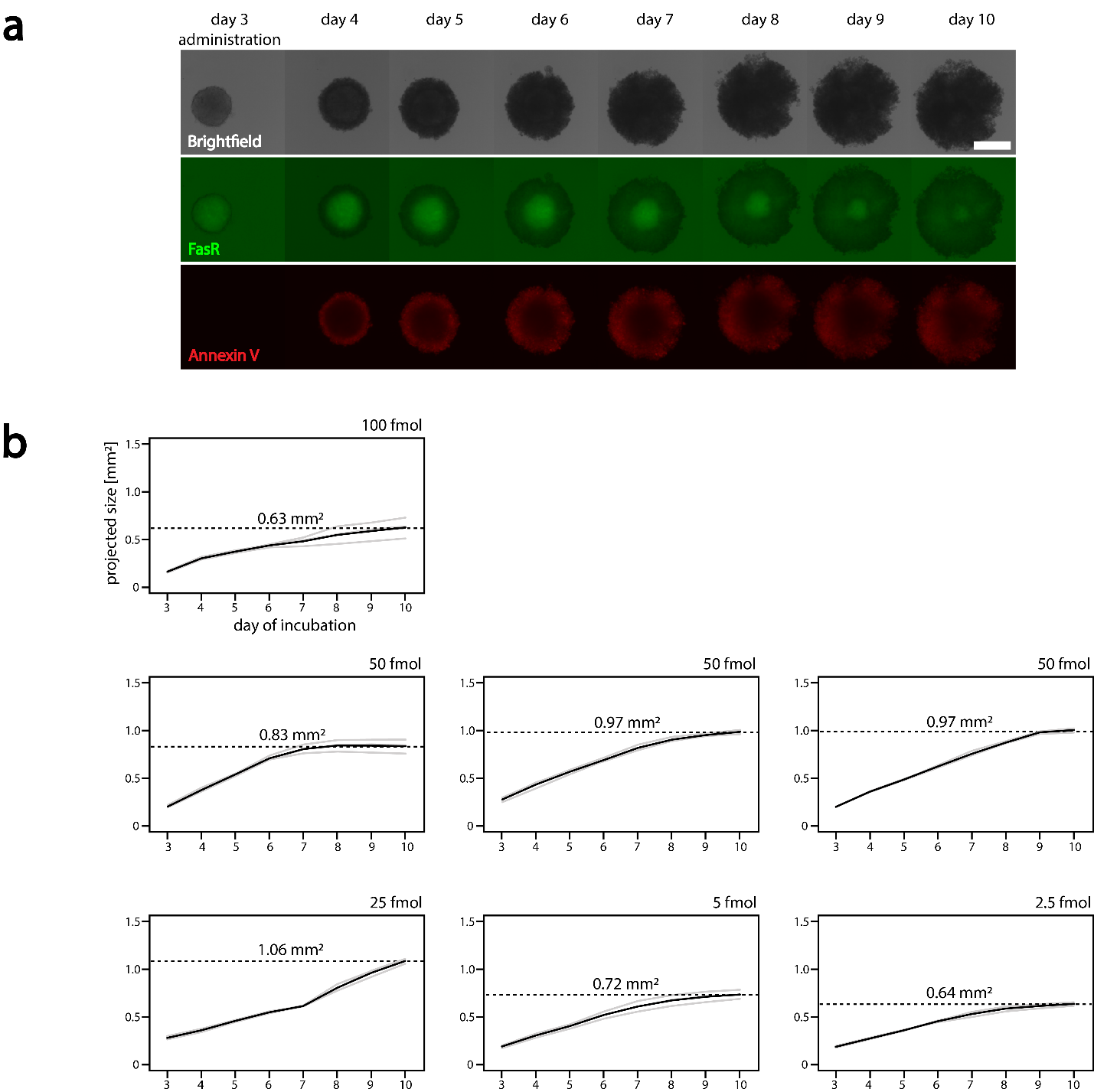


**Figure S23: Development curves of spheroids with wfOF nanoagent** (**a**) Brightfield, GFP (FasR), and Texas Red (Annexin V) images of a spheroid with 50 fmol of wfOF nanoagent over 7 d (from day 3 to day 10). (**b**) Size projections of spheroids incubated with 100 fmol, 50 fmol (triplicate of triplicates), 25 fmol, 5 fmol, and 2.5 fmol nanoagent. Data were also used in Figure 3. The scale bar in (**a**) is 500 µm and holds for all images. Thin grey lines indicate single experiments and thick black lines indicate averages of those (n=3).


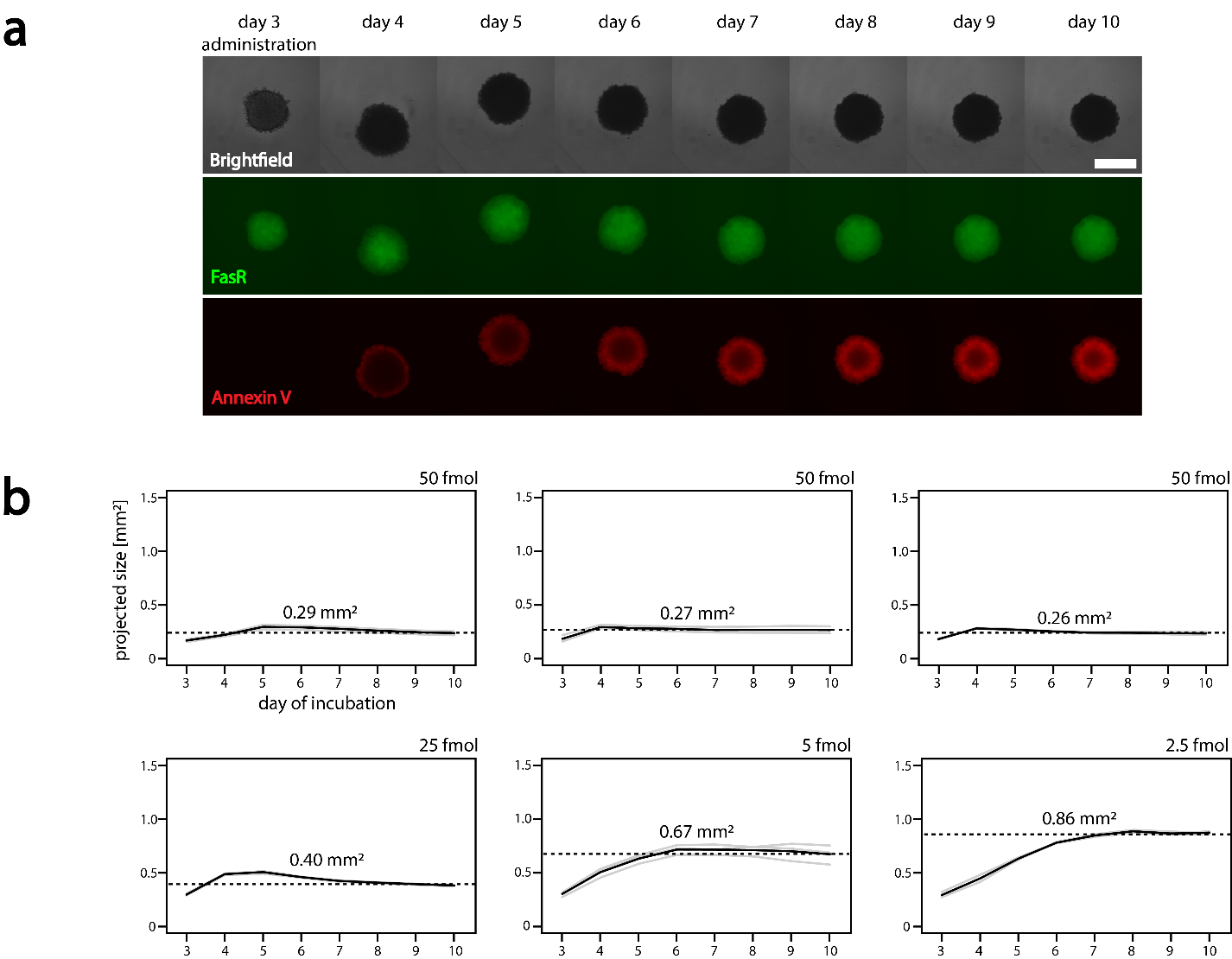


**Figure S24: Development curves of spheroids with rroONF nanoagent** (**a**) Brightfield, GFP (FasR), and Texas Red (Annexin V) images of a spheroid with 50 fmol of rroONF nanoagent over 7 d (from day 3 to day 10). (**b**) Size projections of spheroids incubated with 50 fmol (triplicate of triplicates), 25 fmol, 5 fmol, and 2.5 fmol nanoagent. Data were also used in Figure 3. The scale bar in (**a**) is 500 µm and holds for all images. Thin grey lines indicate single experiments and thick black lines indicate averages of those (n=3).


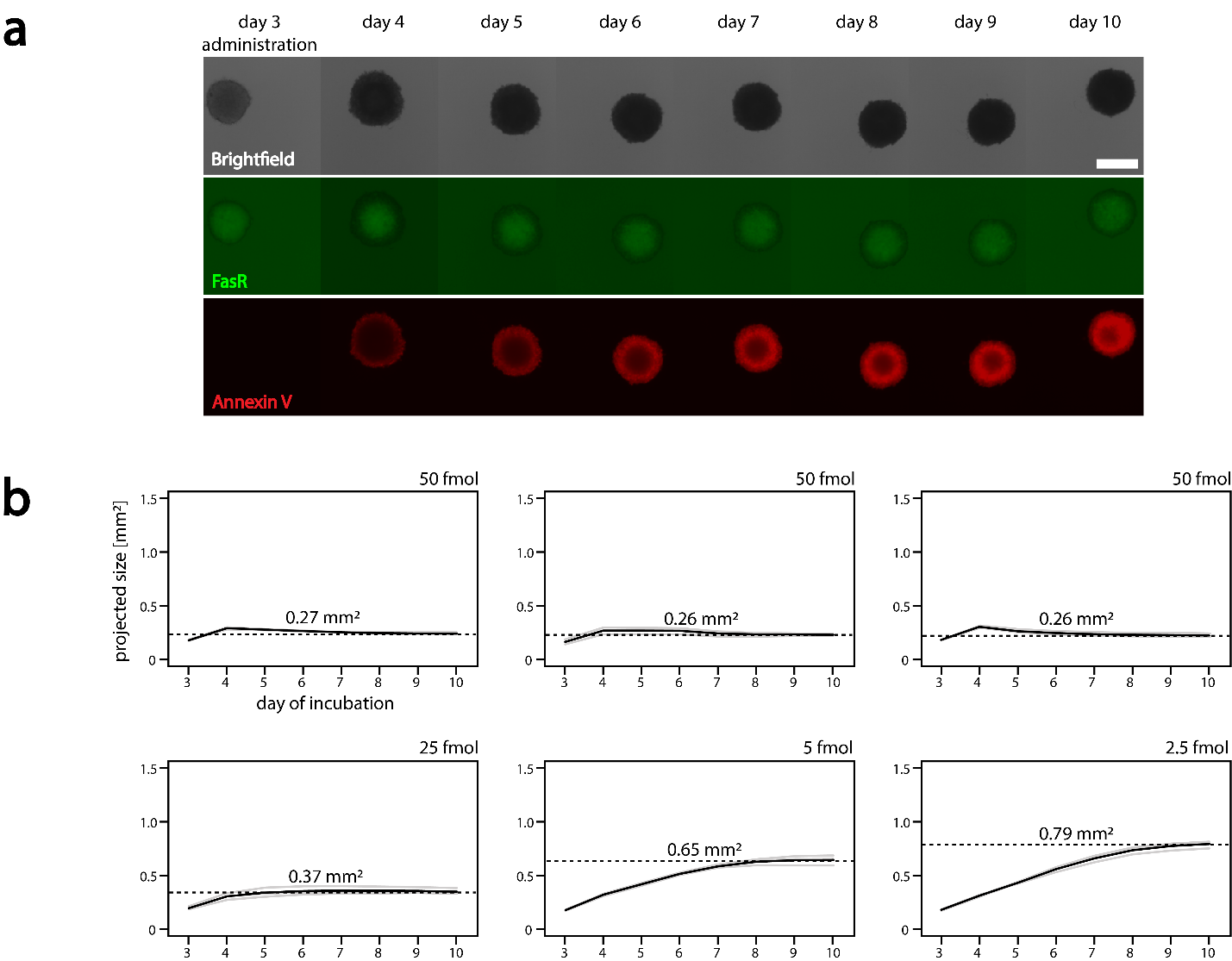


**Figure S25: Development curves of spheroids with miniONF nanoagent** (**a**) Brightfield, GFP (FasR), and Texas Red (Annexin V) images of a spheroid with 50 fmol of miniONF nanoagent over 7 d (from day 3 to day 10). (**b**) Size projections of spheroids incubated with 50 fmol (triplicate of triplicates), 25 fmol, 5 fmol, and 2.5 fmol nanoagent. Data were also used in Figure 3. The scale bar in (**a**) is 500 µm and holds for all images. Thin grey lines indicate single experiments and thick black lines indicate averages of those (n=3).


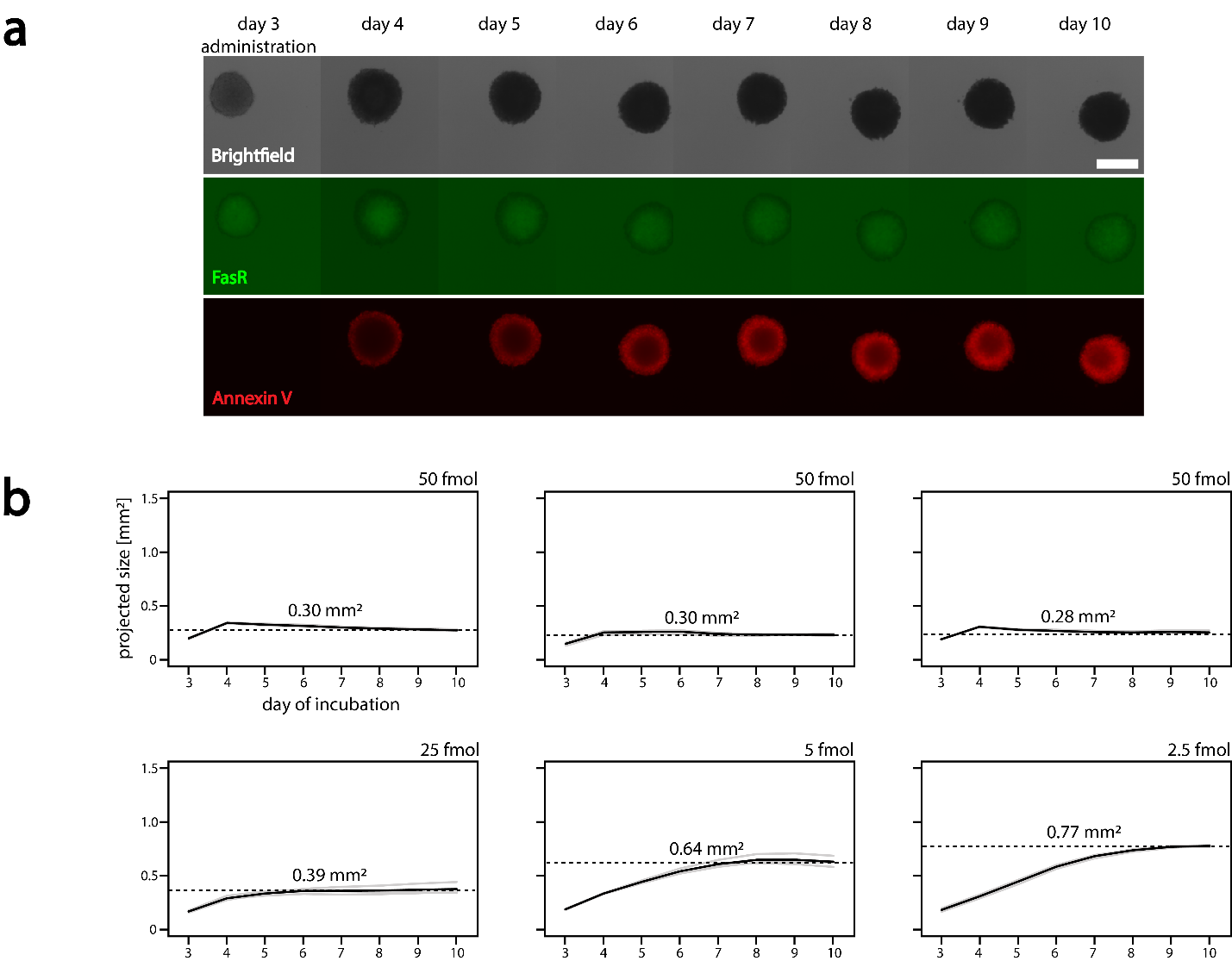


**Figure S26: Development curves of spheroids with wfONF nanoagent** (**a**) Brightfield, GFP (FasR), and Texas Red (Annexin V) images of a spheroid with 50 fmol of wfONF nanoagent over 7 d (from day 3 to day 10). (**b**) Size projections of spheroids incubated with 50 fmol (triplicate of triplicates), 25 fmol, 5 fmol, and 2.5 fmol nanoagent. Data were also used in Figure 3. The Scale bar in (**a**) is 500 µm. Thin grey lines indicate single experiments and thick black lines indicate averages of those (n=3).


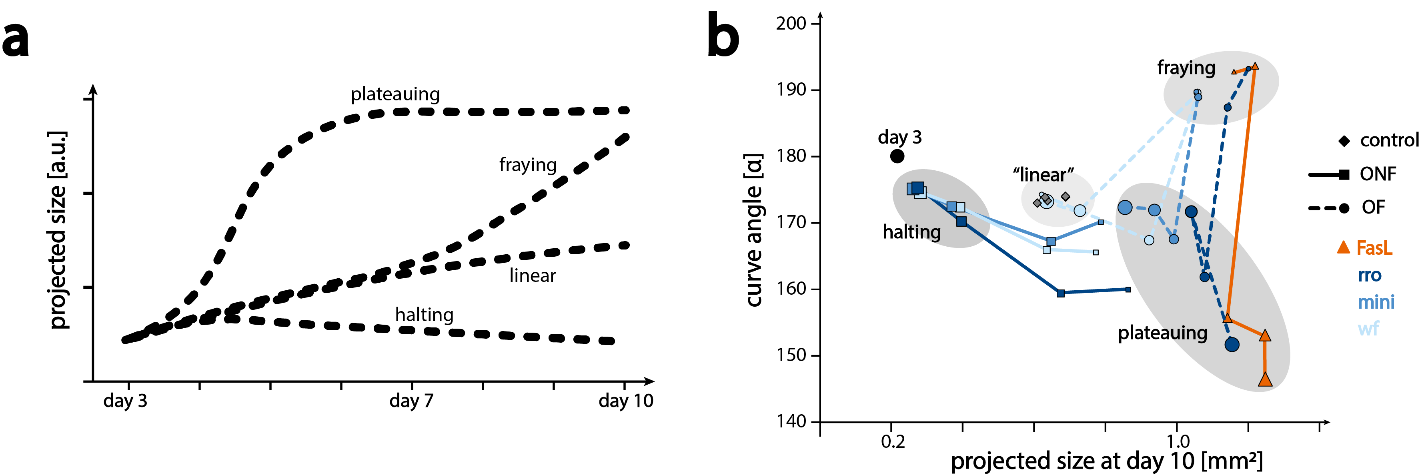


**Figure S27: Pseudo-phase diagram of cancer spheroid behavior** (**a**) Different qualitative behaviors of spheroids, plateauing was observed for large amounts of FasL or OF nanoagents, lower amounts of those led to fraying, then to linear growth, similar to the controls. Halting behavior was observed for larger amounts of ONF nanoagents. (**b**) Pseudo-phase diagram, of spheroid behavior, where the opening angles of the curves are plotted against their final spheroid size (averages from Figures S19-S26). Concentrations of nanoagents are indicated by the size of the respective markers. Several distinct populations are observed. Linear growth is observed for all controls. FasL and OF nanoagents induce plateauing and with lower concentrations, fraying behavior. The halting behavior, induced by ONF nanoagents, is distinctly different from the others.

***
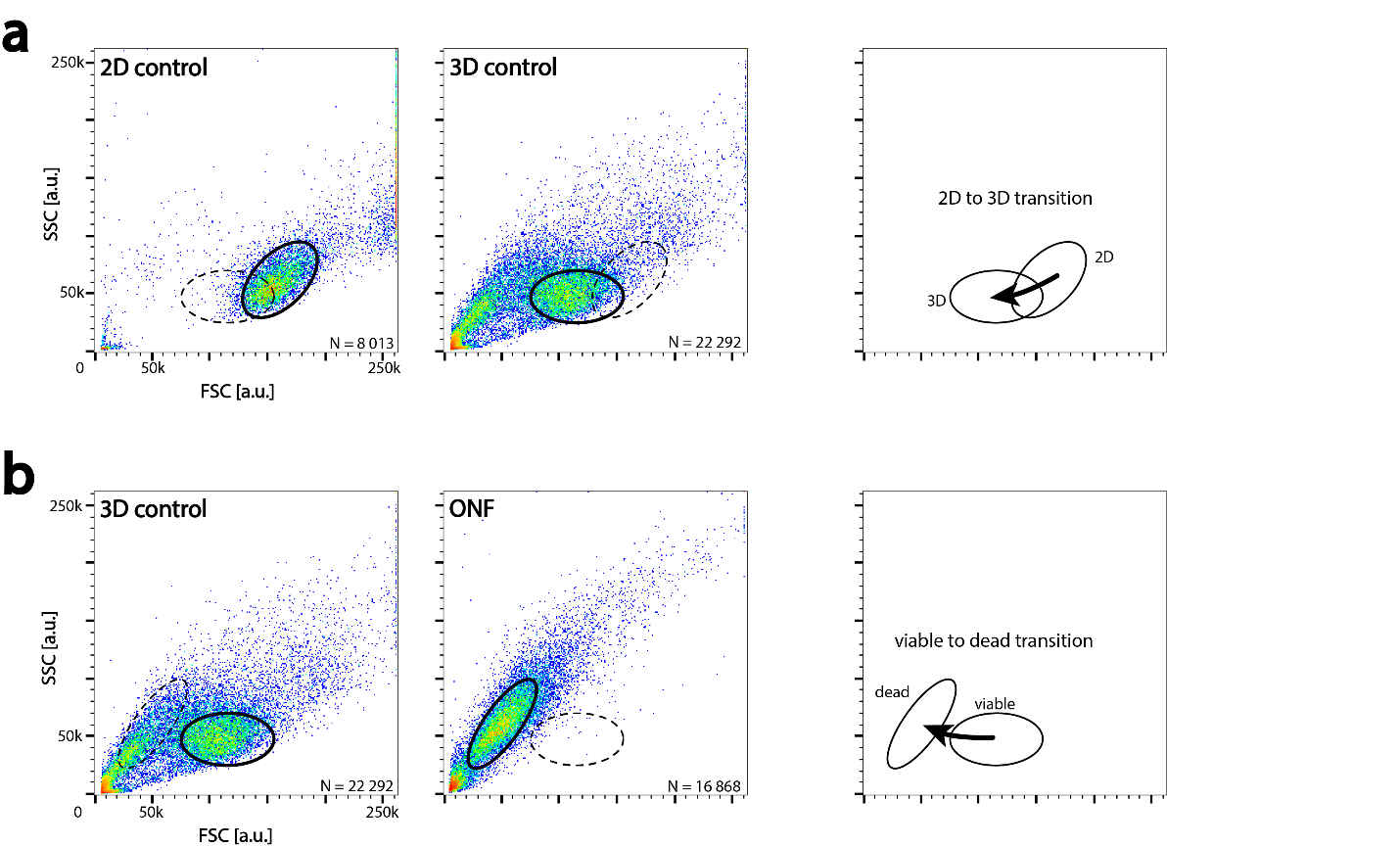
***

**Figure S28: Population shifts in fluorescence activated cell sorting (FACS)** are seen when (**a**) comparing cells cultured in 2D vs. 3D: the 3D control shows a shift in FSC, indicating a size decrease of individual cells cultured in 3D. (**b**) The effectiveness of the respective nanoagent was measured as the ratio between events in viable and dead gates. The higher the ratio of cells in the dead gate compared to the viable gate, the more effective the nanoagent.

***
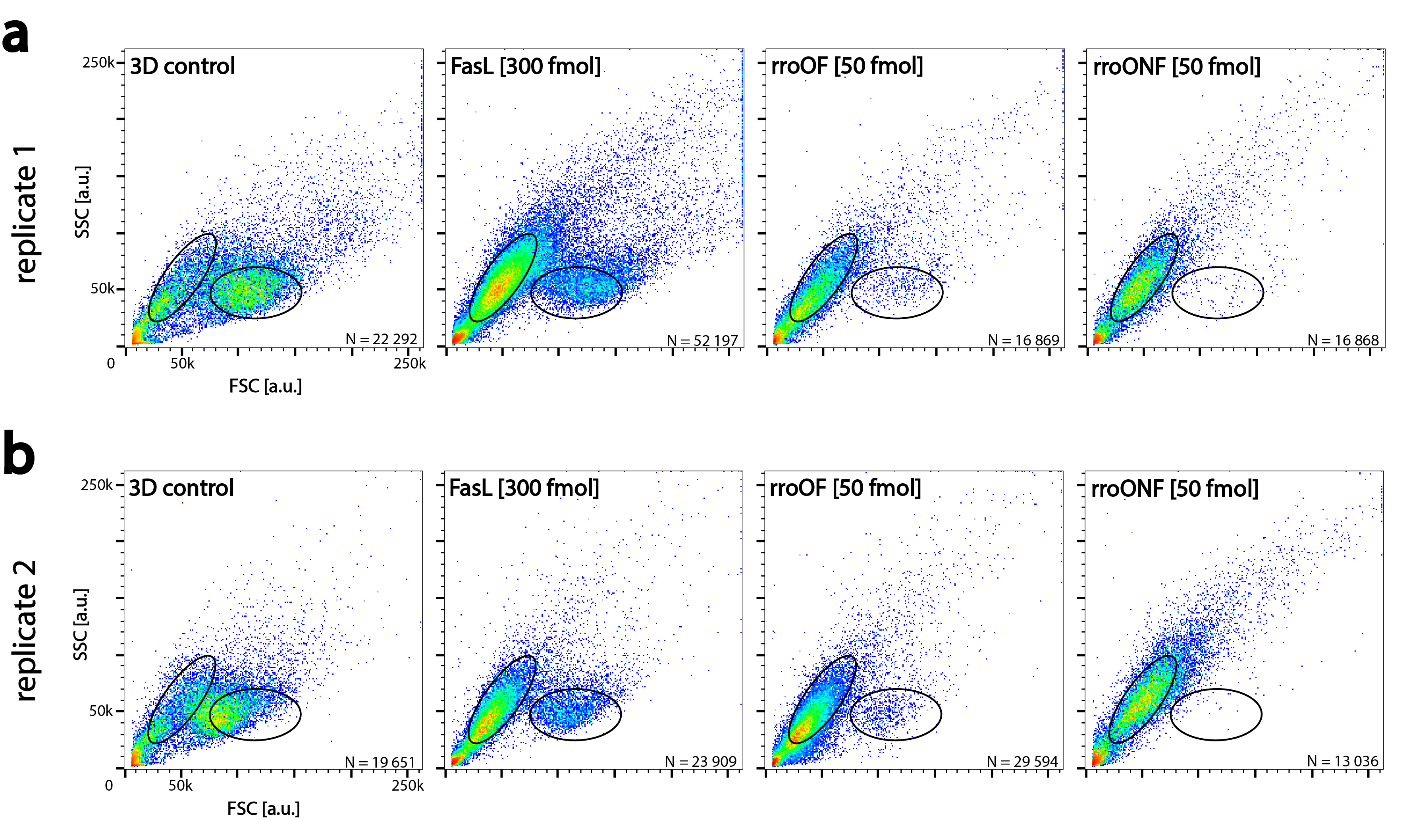
***

**Figure S29: FACS data of dissolved spheroids** in two replicates (**a**) and (**b**). Incubated with 300 fmol FasL, 50 fmol of rroOF or rroONF nanoagent or without addition, as controls. Both replicates, (**a**) and (**b**), show similar distributions depending on which nanoagent was added: The ratio of events in the viable gates to dead gates is largest in the control, and decreases when the spheroids were incubated with FasL. When OF nanoagent was added, the ratio was again lower, and with rroONF nanoagent, nearly no events remained in the viable gate. Data from the same experiment was used for Figure 4.


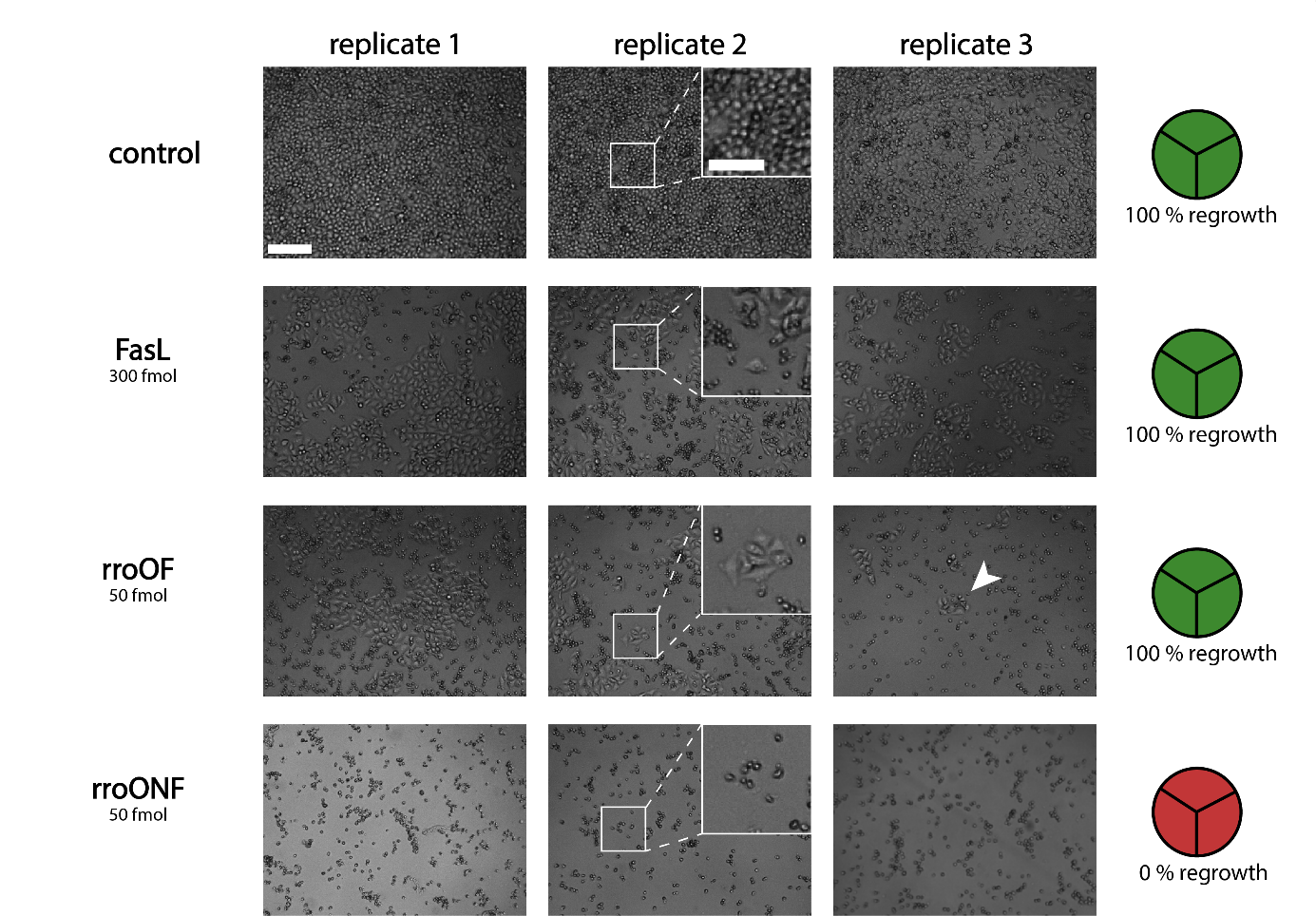


**Figure S30: 2D regrowth of dissolved spheroids** after incubation with the respective additive, dissociation, and seeding on a cell culture-treated dish. Images were taken after 2 d of incubation, and badly visible colonies were marked with a white arrow. rroONF data is the same as in Figure S31. Data was also used in Figure 4. The scale bar is 200 µm and holds for every overview image, and the scale bar in the zoom-in is 100 µm.


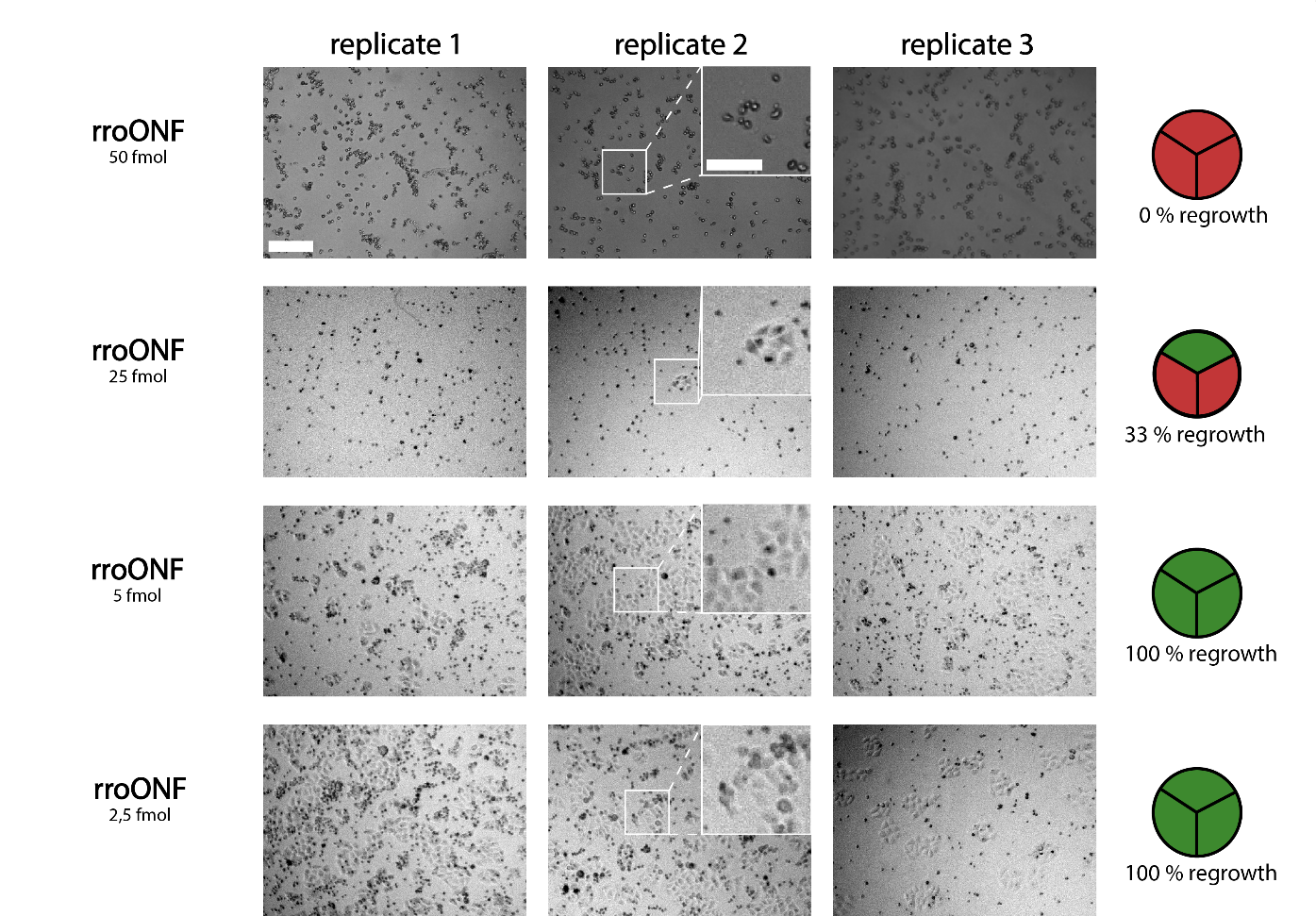


**Figure S31: 2D regrowth of dissolved spheroids, titrating concentrations:** After incubation with the respective nanoagent, dissociation, and seeding on a cell culture-treated dish. Images were taken after 2 d of incubation. rroONF data is the same as in Figure S30, data was also used in Figure 4. The scale bar is 200 µm and holds for every overview image, and the scale bar in the zoom-in is 100 µm.

**Supplementary Note 1: On potential effects of different attachment yields**

For the two kinds of nanoagents, with respect to their attachment strategy (*i.e.* ONF, with neutravidin-biotin; OF, with dsDNA), similar attachment efficiencies were measured. The attachment efficiency for the OF was determined to be approximately 71%[1], and the attachment efficiency for the ONF was determined to be approximately 76%[2]. We suppose the 5% difference here does not have large consequences for the apoptosis efficacy, as we already saw for adherent cells in 2D[2], that dimers of FasL on DNA origami were able to induce a robust apoptosis signal.

Rather than the probability of attachment for each individual FasL, we deem the probability to form a *functional* nanoagent as the relevant parameter (non-functional meaning without or with only one FasL attached). This probability would then be

P_dimer_ = 1-( P_none_ + 6 P_one_) = 1 – ((1-P_attached_)^6^ + 6 (1-P_attached_)^5^ P_attached_)

thus P_ONF_= 99.62 % and P_OF_= 99.07 %, with just 0.55 % difference in probability for the two nanoagent variants.

**Supplementary Note 2: On the mechanisms behind increased apoptosis induction efficacy of ONF nanoagents**

The attachment strategy of FasL to the DNA origami proved far more important for effective apoptosis induction than the structure of the underlying DNA origami. This can be explained by the flexibility of the attachment being larger than the flexibility in positioning. The approximate length of the dsDNA linker of the OF nanoagents is 7 nm, which adds a total of 14 nm margin to the position of any FasL pair on the OF nanoagents. The neutravidin is only 5 nm in size, but also much more voluminous than the dsDNA, which largely restricts its movement and tilt angle on the DNA origami surface, thus decreasing the flexibility.

Further, the dsDNA linker creates two hinge regions, one at its attachment point to the DNA origami and one at the attachment point of the FasL to the dsDNA linker. This increases not only the positional inaccuracy but also the inaccuracy of the FasL angle. As the FasL needs to interact with the FasR on the cells surface in a certain angle, perpendicular to the cells surface[3], this again hinders efficient signaling.

It has also been previously shown that membrane-bound FasL is far more efficient at inducing apoptosis compared to freely diffusing, soluble FasL[2, 4, 5], where soluble FasL often fails to initiate effective apoptotic signaling and may even promote non-apoptotic pathways[6, 7]. The increased flexibility inferred by the dsDNA linker may cause FasL to act like a mixture of soluble and tightly immobilized FasL, inducing apoptosis only to some extent.

**Table S1: rro DNA origami staples**

| name | sequence |
| --- | --- |
| rro_core_001 | TTTTCACTCAAAGGGCGAAAAACCATCACC |
| rro_core_002 | GTCGACTTCGGCCAACGCGCGGGGTTTTTC |
| rro_core_003 | TGCATCTTTCCCAGTCACGACGGCCTGCAG |
| rro_core_004 | TAATCAGCGGATTGACCGTAATCGTAACCG |
| rro_core_005 | AACGCAAAATCGATGAACGGTACCGGTTGA |
| rro_core_006 | AACAGTTTTGTACCAAAAACATTTTATTTC |
| rro_core_007 | TTTACCCCAACATGTTTTAAATTTCCATAT |
| rro_core_008 | TTTAGGACAAATGCTTTAAACAATCAGGTC |
| rro_core_009 | CATCAAGTAAAACGAACTAACGAGTTGAGA |
| rro_core_010 | AATACGTTTGAAAGAGGACAGACTGACCTT |
| rro_core_011 | AGGCTCCAGAGGCTTTGAGGACACGGGTAA |
| rro_core_012 | AGAAAGGAACAACTAAAGGAATTCAAAAAAA |
| rro_core_013 | CAAATCAAGTTTTTTGGGGTCGAAACGTGGA |
| rro_core_014 | CTCCAACGCAGTGAGACGGGCAACCAGCTGCA |
| rro_core_015 | TTAATGAACTAGAGGATCCCCGGGGGGTAACG |
| rro_core_016 | CCAGGGTTGCCAGTTTGAGGGGACCCGTGGGA |
| rro_core_017 | ACAAACGGAAAAGCCCCAAAAACACTGGAGCA |
| rro_core_018 | AACAAGAGGGATAAAAATTTTTAGCATAAAGC |
| rro_core_019 | TAAATCGGGATTCCCAATTCTGCGATATAATG |
| rro_core_020 | CTGTAGCTTGACTATTATAGTCAGTTCATTGA |
| rro_core_021 | ATCCCCCTATACCACATTCAACTAGAAAAATC |
| rro_core_022 | TACGTTAAAGTAATCTTGACAAGAACCGAACT |
| rro_core_023 | GACCAACTAATGCCACTACGAAGGGGGTAGCA |
| rro_core_024 | ACGGCTACAAAAGGAGCCTTTAATGTGAGAAT |
| rro_core_025 | AGCTGATTGCCCTTCAGAGTCCACTATTAAAGGGTGCCGT |
| rro_core_026 | GTATAAGCCAACCCGTCGGATTCTGACGACAGTATCGGCCGCAAGGCG |
| rro_core_027 | TATATTTTGTCATTGCCTGAGAGTGGAAGATT |
| rro_core_028 | GATTTAGTCAATAAAGCCTCAGAGAACCCTCA |
| rro_core_029 | CGGATTGCAGAGCTTAATTGCTGAAACGAGTA |
| rro_core_030 | ATGCAGATACATAACGGGAATCGTCATAAATAAAGCAAAG |
| rro_core_031 | TTTATCAGGACAGCATCGGAACGACACCAACCTAAAACGAGGTCAATC |
| rro_core_032 | ACAACTTTCAACAGTTTCAGCGGATGTATCGG |
| rro_core_033 | AAAGCACTAAATCGGAACCCTAATCCAGTT |
| rro_core_034 | TGGAACAACCGCCTGGCCCTGAGGCCCGCT |
| rro_core_035 | TTCCAGTCGTAATCATGGTCATAAAAGGGG |
| rro_core_036 | GATGTGCTTCAGGAAGATCGCACAATGTGA |
| rro_core_037 | GCGAGTAAAAATATTTAAATTGTTACAAAG |
| rro_core_038 | GCTATCAGAAATGCAATGCCTGAATTAGCA |
| rro_core_039 | AAATTAAGTTGACCATTAGATACTTTTGCG |
| rro_core_040 | GATGGCTTATCAAAAAGATTAAGAGCGTCC |
| rro_core_041 | AATACTGCCCAAAAGGAATTACGTGGCTCA |
| rro_core_042 | TTATACCACCAAATCAACGTAACGAACGAG |
| rro_core_043 | GCGCAGACAAGAGGCAAAAGAATCCCTCAG |
| rro_core_044 | CAGCGAAACTTGCTTTCGAGGTGTTGCTAA |
| rro_core_045 | AGCAAGCGTAGGGTTGAGTGTTGTAGGGAGCC |
| rro_core_046 | CTGTGTGATTGCGTTGCGCTCACTAGAGTTGC |
| rro_core_047 | GCTTTCCGATTACGCCAGCTGGCGGCTGTTTC |
| rro_core_048 | ATATTTTGGCTTTCATCAACATTATCCAGCCA |
| rro_core_049 | TAGGTAAACTATTTTTGAGAGATCAAACGTTA |
| rro_core_050 | AATGGTCAACAGGCAAGGCAAAGAGTAATGTG |
| rro_core_051 | TAAGAGCAAATGTTTAGACTGGATAGGAAGCC |
| rro_core_052 | TCATTCAGATGCGATTTTAAGAACAGGCATAG |
| rro_core_053 | ACACTCATCCATGTTACTTAGCCGAAAGCTGC |
| rro_core_054 | AAACAGCTTTTTGCGGGATCGTCAACACTAAA |
| rro_core_055 | TAAATGAATTTTCTGTATGGGATTAATTTCTT |
| rro_core_056 | CCCGATTTAGAGCTTGACGGGGAAAAAGAATA |
| rro_core_057 | GCCCGAGAGTCCACGCTGGTTTGCAGCTAACT |
| rro_core_058 | CACATTAAAATTGTTATCCGCTCATGCGGGCC |
| rro_core_059 | TCTTCGCTGCACCGCTTCTGGTGCGGCCTTCC |
| rro_core_060 | GAGGGTAGGATTCAAAAGGGTGAGACATCCAA |
| rro_core_061 | TAAATCATATAACCTGTTTAGCTAACCTTTAA |
| rro_core_062 | AATAGTAAACACTATCATAACCCTCATTGTGA |
| rro_core_063 | ATTACCTTTGAATAAGGCTTGCCCAAATCCGC |
| rro_core_064 | GACCTGCTCTTTGACCCCCAGCGAGGGAGTTA |
| rro_core_065 | AAGGCCGCTGATACCGATAGTTGCGACGTTAG |
| rro_core_066 | CCCAGCAGGCGAAAAATCCCTTATAAATCAAGCCGGCG |
| rro_core_067 | TAAATCAAAATAATTCGCGTCTCGGAAACCAGGCAAAGGGAAGG |
| rro_core_068 | GAGACAGCTAGCTGATAAATTAATTTTTGT |
| rro_core_069 | TTTGGGGATAGTAGTAGCATTAAAAGGCCG |
| rro_core_070 | GCTTCAATCAGGATTAGAGAGTTATTTTCA |
| rro_core_071 | CGTTTACCAGACGACAAAGAAGTTTTGCCATAATTCGA |
| rro_core_072 | TGACAACTCGCTGAGGCTTGCATTATACCAAGCGCGATGATAAA |
| rro_core_073 | TCTAAAGTTTTGTCGTCTTTCCAGCCGACAA |
| rro_core_074 | TCAATATCGAACCTCAAATATCAATTCCGAAA |
| rro_core_075 | GCAATTCACATATTCCTGATTATCAAAGTGTA |
| rro_core_076 | AGAAAACAAAGAAGATGATGAAACAGGCTGCG |
| rro_core_077 | ATCGCAAGTATGTAAATGCTGATGATAGGAAC |
| rro_core_078 | CCAATAGCTCATCGTAGGAATCATGGCATCAA |
| rro_core_079 | AGAGAGAAAAAAATGAAAATAGCAAGCAAACT |
| rro_core_080 | GCAAGGCCTCACCAGTAGCACCATGGGCTTGA |
| rro_core_081 | TTGACAGGCCACCACCAGAGCCGCGATTTGTA |
| rro_core_082 | TTAGGATTGGCTGAGACTCCTCAATAACCGAT |
| rro_core_083 | TCCACAGACAGCCCTCATAGTTAGCGTAACGA |
| rro_core_084 | AACGTGGCGAGAAAGGAAGGGAAACCAGTAA |
| rro_core_085 | TCGGCAAATCCTGTTTGATGGTGGACCCTCAA |
| rro_core_086 | AAGCCTGGTACGAGCCGGAAGCATAGATGATG |
| rro_core_087 | CAACTGTTGCGCCATTCGCCATTCAAACATCA |
| rro_core_088 | GCCATCAAGCTCATTTTTTAACCACAAATCCA |
| rro_core_089 | CAACCGTTTCAAATCACCATCAATTCGAGCCA |
| rro_core_090 | CCAACAGGAGCGAACCAGACCGGAGCCTTTAC |
| rro_core_091 | CTTTTGCAGATAAAAACCAAAATAAAGACTCC |
| rro_core_092 | GATGGTTTGAACGAGTAGTAAATTTACCATTA |
| rro_core_093 | TCATCGCCAACAAAGTACAACGGACGCCAGCA |
| rro_core_094 | ATATTCGGAACCATCGCCCACGCAGAGAAGGA |
| rro_core_095 | TAAAAGGGACATTCTGGCCAACAAAGCATC |
| rro_core_096 | ACCTTGCTTGGTCAGTTGGCAAAGAGCGGA |
| rro_core_097 | ATTATCATTCAATATAATCCTGACAATTAC |
| rro_core_098 | CTGAGCAAAAATTAATTACATTTTGGGTTA |
| rro_core_099 | TATAACTAACAAAGAACGCGAGAACGCCAA |
| rro_core_100 | CATGTAATAGAATATAAAGTACCAAGCCGT |
| rro_core_101 | TTTTATTTAAGCAAATCAGATATTTTTTGT |
| rro_core_102 | TTAACGTCTAACATAAAAACAGGTAACGGA |
| rro_core_103 | ATACCCAACAGTATGTTAGCAAATTAGAGC |
| rro_core_104 | CAGCAAAAGGAAACGTCACCAATGAGCCGC |
| rro_core_105 | CACCAGAAAGGTTGAGGCAGGTCATGAAAG |
| rro_core_106 | TATTAAGAAGCGGGGTTTTGCTCGTAGCAT |
| rro_core_107 | TCAACAGTTGAAAGGAGCAAATGAAAAATCTAGAGATAGA |
| rro_core_108 | TCAAATATAACCTCCGGCTTAGGTAACAATTTCATTTGAAGGCGAATT |
| rro_core_109 | GTAAAGTAATCGCCATATTTAACAAAACTTTT |
| rro_core_110 | TATCCGGTCTCATCGAGAACAAGCGACAAAAG |
| rro_core_111 | TTAGACGGCCAAATAAGAAACGATAGAAGGCT |
| rro_core_112 | CGTAGAAAATACATACCGAGGAAACGCAATAAGAAGCGCA |
| rro_core_113 | GCGGATAACCTATTATTCTGAAACAGACGATTGGCCTTGAAGAGCCAC |
| rro_core_114 | TCACCAGTACAAACTACAACGCCTAGTACCAG |
| rro_core_115 | ACCCTTCTGACCTGAAAGCGTAAGACGCTGAG |
| rro_core_116 | AGCCAGCAATTGAGGAAGGTTATCATCATTTT |
| rro_core_117 | GCGGAACATCTGAATAATGGAAGGTACAAAAT |
| rro_core_118 | CGCGCAGATTACCTTTTTTAATGGGAGAGACT |
| rro_core_119 | ACCTTTTTATTTTAGTTAATTTCATAGGGCTT |
| rro_core_120 | AATTGAGAATTCTGTCCAGACGACTAAACCAA |
| rro_core_121 | GTACCGCAATTCTAAGAACGCGAGTATTATTT |
| rro_core_122 | ATCCCAATGAGAATTAACTGAACAGTTACCAG |
| rro_core_123 | AAGGAAACATAAAGGTGGCAACATTATCACCG |
| rro_core_124 | TCACCGACGCACCGTAATCAGTAGCAGAACCG |
| rro_core_125 | CCACCCTCTATTCACAAACAAATACCTGCCTA |
| rro_core_126 | TTTCGGAAGTGCCGTCGAGAGGGTGAGTTTCG |
| rro_core_127 | CTTTAGGGCCTGCAACAGTGCCAATACGTG |
| rro_core_128 | CTACCATAGTTTGAGTAACATTTAAAATAT |
| rro_core_129 | CATAAATCTTTGAATACCAAGTGTTAGAAC |
| rro_core_130 | CCTAAATCAAAATCATAGGTCTAAACAGTA |
| rro_core_131 | ACAACATGCCAACGCTCAACAGTCTTCTGA |
| rro_core_132 | GCGAACCTCCAAGAACGGGTATGACAATAA |
| rro_core_133 | AAAGTCACAAAATAAACAGCCAGCGTTTTA |
| rro_core_134 | AACGCAAAGATAGCCGAACAAACCCTGAAC |
| rro_core_135 | TCAAGTTTCATTAAAGGTGAATATAAAAGA |
| rro_core_136 | TTAAAGCCAGAGCCGCCACCCTCGACAGAA |
| rro_core_137 | GTATAGCAAACAGTTAATGCCCAATCCTCA |
| rro_core_138 | AGGAACCCATGTACCGTAACACTTGATATAA |
| rro_core_139 | GCACAGACAATATTTTTGAATGGGGTCAGTA |
| rro_core_140 | TTAACACCAGCACTAACAACTAATCGTTATTA |
| rro_core_141 | ATTTTAAAATCAAAATTATTTGCACGGATTCG |
| rro_core_142 | CCTGATTGCAATATATGTGAGTGATCAATAGT |
| rro_core_143 | GAATTTATTTAATGGTTTGAAATATTCTTACC |
| rro_core_144 | AGTATAAAGTTCAGCTAATGCAGATGTCTTTC |
| rro_core_145 | CTTATCATTCCCGACTTGCGGGAGCCTAATTT |
| rro_core_146 | GCCAGTTAGAGGGTAATTGAGCGCTTTAAGAA |
| rro_core_147 | AAGTAAGCAGACACCACGGAATAATATTGACG |
| rro_core_148 | GAAATTATTGCCTTTAGCGTCAGACCGGAACC |
| rro_core_149 | GCCTCCCTCAGAATGGAAAGCGCAGTAACAGT |
| rro_core_150 | GCCCGTATCCGGAATAGGTGTATCAGCCCAAT |
| rro_core_151 | AGATTAGAGCCGTCAAAAAACAGAGGTGAGGCCTATTAGT |
| rro_core_152 | GTGATAAAAAGACGCTGAGAAGAGATAACCTTGCTTCTGTTCGGGAGA |
| rro_core_153 | GTTTATCAATATGCGTTATACAAACCGACCGT |
| rro_core_154 | GCCTTAAACCAATCAATAATCGGCACGCGCCT |
| rro_core_155 | GAGAGATAGAGCGTCTTTCCAGAGGTTTTGAA |
| rro_core_156 | GTTTATTTTGTCACAATCTTACCGAAGCCCTTTAATATCA |
| rro_core_157 | CAGGAGGTGGGGTCAGTGCCTTGAGTCTCTGAATTTACCGGGAACCAG |
| rro_core_158 | CCACCCTCATTTTCAGGGATAGCAACCGTACT |
| rro_core_159 | CTTTAATGCGCGAACTGATAGCCCCACCAG |
| rro_core_160 | CAGAAGATTAGATAATACATTTGTCGACAA |
| rro_core_161 | CTCGTATTAGAAATTGCGTAGATACAGTAC |
| rro_core_162 | CTTTTACAAAATCGTCGCTATTAGCGATAG |
| rro_core_163 | CTTAGATTTAAGGCGTTAAATAAAGCCTGT |
| rro_core_164 | TTAGTATCACAATAGATAAGTCCACGAGCA |
| rro_core_165 | TGTAGAAATCAAGATTAGTTGCTCTTACCA |
| rro_core_166 | ACGCTAACACCCACAAGAATTGAAAATAGC |
| rro_core_167 | AATAGCTATCAATAGAAAATTCAACATTCA |
| rro_core_168 | ACCGATTGTCGGCATTTTCGGTCATAATCA |
| rro_core_169 | AAATCACCTTCCAGTAAGCGTCAGTAATAA |
| rro_core_170 | GTTTTAACTTAGTACCGCCACCCAGAGCCA |
| rro_anchor_01 | CATTCTCCTATTACTACCTTGTGTCGTGACGAGAAACACCAAATTTCAACTTTAAT |
| rro_anchor_02 | CATTCTCCTATTACTACCGCGATCGGCAATTCCACACAACAGGTGCCTAATGAGTG |
| rro_anchor_03 | CATTCTCCTATTACTACCCACCCTCAGAAACCATCGATAGCATTGAGCCATTTGGGAA |
| rro_anchor_04 | CATTCTCCTATTACTACCAACAATAACGTAAAACAGAAATAAAAATCCTTTGCCCGAA |
| rro_anchor_05 | CATTCTCCTATTACTACCATTAAGTTTACCGAGCTCGAATTCGGGAAACCTGTCGTGC |
| rro_anchor_06 | CATTCTCCTATTACTACCCACCCTCAGAAACCATCGATAGCATTGAGCCATTTGGGAA |
| rro_anchor_07 | CATTCTCCTATTACTACCATAAGGGAACCGGATATTCATTACGTCAGGACGTTGGGAA |
| rro_anchor_08 | CATTCTCCTATTACTACCAGCCACCACTGTAGCGCGTTTTCAAGGGAGGGAAGGTAAA |
|  |  |
| rro_FasL_handle_01 | CGAAAGACTTTGATAAGAGGTCATATTTCGCA TT TTCATTCTCCTATTACTACC |
| rro_FasL_handle_02 | TGTAGCCATTAAAATTCGCATTAAATGCCGGA TT TTCATTCTCCTATTACTACC |
| rro_FasL_handle_03 | TTGCTCCTTTCAAATATCGCGTTTGAGGGGGT TT TTCATTCTCCTATTACTACC |
| rro_FasL_handle_04 | GTAATAAGTTAGGCAGAGGCATTTATGATATT TT TTCATTCTCCTATTACTACC |
| rro_FasL_handle_05 | TTATTACGAAGAACTGGCATGATTGCGAGAGG TT TTCATTCTCCTATTACTACC |
| rro_FasL_handle_06 | TTCTACTACGCGAGCTGAAAAGGTTACCGCGC TT TTCATTCTCCTATTACTACC |
| rro_biotin_handle_01 | CGAAAGACTTTGATAAGAGGTCATATTTCGCA TT [biotin] |
| rro_biotin_handle_02 | TGTAGCCATTAAAATTCGCATTAAATGCCGGA TT [biotin] |
| rro_biotin_handle_03 | TTGCTCCTTTCAAATATCGCGTTTGAGGGGGT TT [biotin] |
| rro_biotin_handle_04 | GTAATAAGTTAGGCAGAGGCATTTATGATATT TT [biotin] |
| rro_biotin_handle_05 | TTATTACGAAGAACTGGCATGATTGCGAGAGG TT [biotin] |
| rro_biotin_handle_06 | TTCTACTACGCGAGCTGAAAAGGTTACCGCGC TT [biotin] |
| rro_FISH_handle_01 | CGAAAGACTTTGATAAGAGGTCATATTTCGCA GCATTCTTTCTTGAGGAGGGCAGCAAACGGGAAGAG |
| rro_FISH_handle_02 | TGTAGCCATTAAAATTCGCATTAAATGCCGGA GCATTCTTTCTTGAGGAGGGCAGCAAACGGGAAGAG |
| rro_FISH_handle_03 | TTGCTCCTTTCAAATATCGCGTTTGAGGGGGT GCATTCTTTCTTGAGGAGGGCAGCAAACGGGAAGAG |
| rro_FISH_handle_04 | GTAATAAGTTAGGCAGAGGCATTTATGATATT GCATTCTTTCTTGAGGAGGGCAGCAAACGGGAAGAG |
| rro_FISH_handle_05 | TTATTACGAAGAACTGGCATGATTGCGAGAGG GCATTCTTTCTTGAGGAGGGCAGCAAACGGGAAGAG |
| rro_FISH_handle_06 | TTCTACTACGCGAGCTGAAAAGGTTACCGCGC GCATTCTTTCTTGAGGAGGGCAGCAAACGGGAAGAG |

**Table S2: mini DNA origami staples**

| name | sequence |
| --- | --- |
| mini_core_01 | ACTCTCGGGTTAAAGAGCACCATCCGGCGGC |
| mini_core_02 | ATCACTCCGCGAACAGTTTCACTGGTGCATAG |
| mini_core_03 | AGCCCGACTAGCTAATAAGCTCCTGAAACAAGTGGCGCAGTGCAGTA |
| mini_core_04 | CAGACGGTATTTGCCGTCAAATGGAGTCTGT |
| mini_core_05 | GATCGCTATATGTTCTATACCCACGTTAAGTT |
| mini_core_06 | CCTGACACCCGAGCATGTTACATTGGGAGCA |
| mini_core_07 | TGTCCGTATGGAGATATAGAACCCTTTCAGAG |
| mini_core_08 | AGGACCCGCCACGCCCTCGCTGCCATTATAC |
| mini_core_09 | GCTTTGAGCTCTCCTGTGTTGTGCGGGTTAGT |
| mini_core_10 | CGCCGGTCTCAGAAGGCCCAAACAGTGTATATCGAATCGCGGAAGTCT |
| mini_core_11 | GCTCCGTGAAGCAGCCGTGCTCCATCTTCGAT |
| mini_core_12 | TCGGGAGGAAGGACACTGTTATCCGTCCGGC |
| mini_core_13 | ATATTCATGGATCCAACCAATTTATTGGAGCT |
| mini_core_14 | CGTTTGACGAAGCTTGATTTAAGGCTTACCC |
| mini_core_15 | TGTGCCGGGAGTATTCCGATGAAAGGTATGT |
| mini_core_16 | GTTGATGCCTCTAGGTACGGATGGTTCAAAG |
| mini_core_17 | CTGCTCGCACGATCGATGGCTGATTAGTGCGG |
| mini_core_18 | TGGAGTTCGTCCGCATGGAGGGCCGTTCTTA |
| mini_core_19 | CGCCATATAGAGAACTGGTTCATTTTGCCAGC |
| mini_core_20 | ATTGGCCATTTACGGGACGCCGCACCGTACT |
|  |  |
| mini_FasL_handle_01 | GATCTACCAGTCATCGTCGTGCAATAACACGG TT TTCATTCTCCTATTACTACC |
| mini_FasL_handle_02 | CTTAAGCCATTGTTCAGGGAGTACAGGCTTG TT TTCATTCTCCTATTACTACC |
| mini_FasL_handle_03 | ACCAAGAACTCCGCTTGCAGAGGCAAAGGTT TT TTCATTCTCCTATTACTACC |
| mini_FasL_handle_04 | TACTTCAGTATCAGTAGTCCCTAAGGCTATGT TT TTCATTCTCCTATTACTACC |
| mini_FasL_handle_05 | GAATTTGACACGGCAGACATCGCGACTGACGC TT TTCATTCTCCTATTACTACC |
| mini_FasL_handle_06 | AATGACGTACGAGGGAATCCACTCCCACATGC TT TTCATTCTCCTATTACTACC |
| mini_biotin_handle_01 | GATCTACCAGTCATCGTCGTGCAATAACACGG TT [biotin] |
| mini_biotin_handle_02 | CTTAAGCCATTGTTCAGGGAGTACAGGCTTG TT [biotin] |
| mini_biotin_handle_03 | ACCAAGAACTCCGCTTGCAGAGGCAAAGGTT TT [biotin] |
| mini_biotin_handle_04 | TACTTCAGTATCAGTAGTCCCTAAGGCTATGT TT [biotin] |
| mini_biotin_handle_05 | GAATTTGACACGGCAGACATCGCGACTGACGC TT [biotin] |
| mini_biotin_handle_06 | AATGACGTACGAGGGAATCCACTCCCACATGC TT [biotin] |
| mini_FISH_handle_01 | GATCTACCAGTCATCGTCGTGCAATAACACGG GCATTCTTTCTTGAGGAGGGCAGCAAACGGGAAGAG |
| mini_FISH_handle_02 | CTTAAGCCATTGTTCAGGGAGTACAGGCTTG GCATTCTTTCTTGAGGAGGGCAGCAAACGGGAAGAG |
| mini_FISH_handle_03 | ACCAAGAACTCCGCTTGCAGAGGCAAAGGTT GCATTCTTTCTTGAGGAGGGCAGCAAACGGGAAGAG |
| mini_FISH_handle_04 | TACTTCAGTATCAGTAGTCCCTAAGGCTATGT GCATTCTTTCTTGAGGAGGGCAGCAAACGGGAAGAG |
| mini_FISH_handle_05 | GAATTTGACACGGCAGACATCGCGACTGACGC GCATTCTTTCTTGAGGAGGGCAGCAAACGGGAAGAG |
| mini_FISH_handle_06 | AATGACGTACGAGGGAATCCACTCCCACATGC GCATTCTTTCTTGAGGAGGGCAGCAAACGGGAAGAG |

**Table S3: wf DNA origami staples**

| name | sequence |
| --- | --- |
| WF_core_001 | CGCCGCCAGCATTGACACCCCCCGTTCAGCCC |
| WF_core_002 | GGTTTGGCTCTTAGGGGAACCACCACCAGAGC |
| WF_core_003 | CCCTCAGAGCCGCCACCACACCGGAACCAGA |
| WF_core_004 | TCAGACGATTGGCCTTGCCACCCTCAGAGCCACCA |
| WF_core_005 | CGCCACCCTCAGAACCGATATTCACAAAC |
| WF_core_006 | TGGCTCCGCCTCCCTCAGAGC |
| WF_core_007 | GCCACCACCGGAAATCGGCATTTTCGG |
| WF_core_008 | TTTTCATAATCAAAATACTGTGAAGACGC |
| WF_core_009 | TTGGAAGGTCAGAATTAGCGTTTGCCATC |
| WF_core_010 | TCATAGCCCCCTTCCGTAATCAGTAG |
| WF_core_011 | ACTGTAGCGCGTTTTCTTTGATGATACAG |
| WF_core_012 | GTGCCTTGAGTAACAGTGTTTGCCTTTAGCGTCAG |
| WF_core_013 | CGACAGAATCAAGAGCACCATTACCA |
| WF_core_014 | ACCATCGATAGCAGCAAGCTGTCAACTGGGTT |
| WF_core_015 | AAGTAGGAGTTAAAGCAAACGTCACCAATGAA |
| WF_core_016 | TTAGCAAGGCCGGAATTATCACCGTC |
| WF_core_017 | CAGCAAAATCACCAGTCCCGTATAAACAGTTA |
| WF_core_018 | GTATCACCGTACTCAGATTTGGGAATTAGAGC |
| WF_core_019 | ACCGACTTGAGCCCATTCAACCGATT |
| WF_core_020 | TTATTCATTAAAGGTGTCCTTAGTTACTT |
| WF_core_021 | ACTCCTTATTACGTAAATATTGACGGAAA |
| WF_core_022 | GAGGGAGGGAAGGTTTGTCACAATCA |
| WF_core_023 | AAAGACAAAAGGGCGAGAGGTTTAGTACC |
| WF_core_024 | CCCTCAGAGCCACCACCCTCAATGGTTTACCAGCGCC |
| WF_core_025 | ATAGAAAATTCATTTTTCAGGGATAGCAAGCC |
| WF_core_026 | GGAATAAGTTTATCAGTATGTTAGCA |
| WF_core_027 | CAATAGGAACCCATGTACGCAAAGACACCAC |
| WF_core_028 | AACATATAAAAGAAACCGTAACACTGAGTTTCGTC |
| WF_core_029 | CCAAGTCGTATGGCTACATACATAAAGGTGGC |
| WF_core_030 | AACGTAGAAAATATCACCCTTCTTTCCGT |
| WF_core_031 | TAAGTAGACGCTACGGTGGCATGATTAAG |
| WF_core_032 | GATGCTACCGGTTCGTGTGCGGTGGCGTATGA |
| WF_core_033 | GTCAATGAAGTCTCCTCCTCTCCATGAAA |
| WF_core_034 | CAGTAGCACCAGATGGAGCTGGAGTAGTC |
| WF_core_035 | TCAAGATTGTTGATGGCGGTACCACATAC |
| WF_core_036 | TCTGTGGCAGCTTAGTTTCCCGCAGA |
| WF_core_037 | GTCTTACAAATGTAAAGTCCTGAACATTTACCTTC |
| WF_core_038 | GCACACCCCCTGCTAACCATACTAACTTT |
| WF_core_039 | CTAAGCCAGTATTATGCGATTGGTGA |
| WF_core_040 | ACCAGTACAAACTACAACTGTAACGATAGGCAAA |
| WF_core_041 | ACGGGTCGTACGCGCCTGTAGCATTCCACAGA |
| WF_core_042 | ATCTGCATCGCAACAATGCACTTTAT |
| WF_core_043 | CAGCCCTCATAGTTAGCGCGTCCGAGTTGTT |
| WF_core_044 | TACTCCGTGGTTACTTTAACGATCTAAAGTTT |
| WF_core_045 | TTTTCTGTATGGGCTCGTAAGAGATAGGA |
| WF_core_046 | AATGAGAATAACATTTAAAAAGGCCGTAA |
| WF_core_047 | TATCCAGCTGAACGGTGTTTAGTATACCC |
| WF_core_048 | ATGGTAATCCCTTATTACTTTTCTCCATTTTAGC |
| WF_core_049 | TCCAACAACCATTCATCTGAGGGCCC |
| WF_core_050 | TGCCTTTTGACTCATCTCACGCTGCGCGT |
| WF_core_051 | CGCCGCTACAGGGATATGATTTCTGCTTG |
| WF_core_052 | GGCGGCCATAGTCGGCCTGTTAAGTG |
| WF_core_053 | CGCCTACTGCGCTCGCTCAGCGATCC |
| WF_core_054 | GACCGCTGCGCCTTATGCAGCTCCCCACTAGA |
| WF_core_055 | GAGGAACTCTGGTAGGCGGTGCTACAGAG |
| WF_core_056 | TTCTTGAAGTGGTGGCGCCGCGCTTAATG |
| WF_core_057 | AACCACCACACCCCTTGATCCGGCAAACA |
| WF_core_058 | AACCACCGCTGGTAGCCGCTAGGGCGCTG |
| WF_core_059 | CGAAAGGAGCGGGCGCTCAGTGGAACGAA |
| WF_core_060 | CGTGGCGAGAAAGTGCCGTAAAGCAC |
| WF_core_061 | AACTCACGTTAAGGGAGAAAGCCGGCGAA |
| WF_core_062 | TTTAGAGCTTGACGGGATGAGTAAACTTGGTC |
| WF_core_063 | GCGGCATCAGCACCTTTAAAGGGAGCCCCCGA |
| WF_core_064 | TAAATCGGAACCCTGGAACCTCTTAC |
| WF_core_065 | GTGCCCGATCAAGAATCTCGATAACT |
| WF_core_066 | TCATTATGGTGAAAGTGTCGCCTTGCGTATAATAT |
| WF_core_067 | TGTCCATATTGGCCGGTAGTGATCTTATT |
| WF_core_068 | CAAAAAATACGCCATCAACGGTGGTA |
| WF_core_069 | TATCCAGTGATTTCTGGTTATAGGTACATTGA |
| WF_core_070 | GATGCCATTGGGATATCACGTTTAAATCAAAA |
| WF_core_071 | AACAAGGGTGAACACTTCAAAATGTTCTTTAC |
| WF_core_072 | GCAACTGACTGAAATGCCACTTGTGCTTATT |
| WF_core_073 | TTTCTTTACGGTCGTTTTGCTAAACAACT |
| WF_core_074 | AAGGCCGGATAAAATCCCATATCACCAGCTCA |
| WF_core_075 | TTCAACAGTTTCAGCGAAGAATGTGAATA |
| WF_core_076 | CATTCATCAGGCGGGCAAGGCTCCAAAAGGAGC |
| WF_core_077 | CTTTAATTGTATCGGTCGAAATTCCGGATGAG |
| WF_core_078 | CCGTCTTTCATTGCCATAAGAGCGATGAAAA |
| WF_core_079 | CTGGTGAAACTCACCCTCATGGAAAACGGTGT |
| WF_core_080 | CGTTTCAGTTTGCGCGAATAT |
| WF_core_081 | TCGTGGTATTCACTCCTTATCAGCTTGCTTTCG |
| WF_core_082 | AGGTGAATTTCGTGAGAAACTGCCGGAAATCG |
| WF_core_083 | ATGTGTAGCAAAAGGCCAGCA |
| WF_core_084 | CACGCCACATCTTAGGGATTGGCTGA |
| WF_core_085 | AAAGGCCAGGAACGTTTTCACCGTAA |
| WF_core_086 | TAGGGAAATAGGCCAGCGTAAAAAGGCCGCGTTGC |
| WF_core_087 | TTAAGCATTCTGCCGATTCTCAATAAACCCTT |
| WF_core_088 | GACGAAAAACATAGGGGCGAAGAAGT |
| WF_core_089 | TTGCCCATGGTGAAAACGCATGGAAGCCATC |
| WF_core_090 | TGACAGCTCGAGGCTTTGAACCTGAATCGCCA |
| WF_core_091 | ACAAACGGCATGACGATATCAAATTACGCCCC |
| WF_core_092 | TGGCGTTTTTCCATAGGCGTACTGTTGTAATTCA |
| WF_core_093 | GCCCTGCCACTCATCGCATCCGCCCCCCTGACGAGCA |
| WF_core_094 | ACAGGAGTCCAAGCGAGCTGGATTCTCACCAA |
| WF_core_095 | TCACAAAAATCGACGCTCGTCATTACTGGATCTATCA |
| WF_core_096 | AAATCCAGATGGAGTTCTGAGAAGTCAGAGGTGGCGAAACCC |
| WF_core_097 | GACAGGACTATAAAGATACCAGCGGCAACCGAGCGTTCTGAAC |
| WF_core_098 | TAAAAAACGCCCGAAGTTTTAAATCA |
| WF_core_099 | ATCTAAAGTATATTTTTGGTCATGAG |
| WF_core_100 | TTTTAAATTAAAAATGGGCGTTTCCCCCTGGA |
| WF_core_101 | AGCTCCCTCGTGCGCTATCTTCACCTAGATCC |
| WF_core_102 | ATTATCAAAAAGGTCCTTTGATCTTTT |
| WF_core_103 | CTACGGGGTCTGAGGTGGTTTTTTTGT |
| WF_core_104 | AAGGATCTCAAGAAGACTCCTGTTCCGACCCT |
| WF_core_105 | GCCGCTTACCGGATACATTACGCGCAGAAAAA |
| WF_core_106 | TTGCAAGCAGCAGTACCTTCGGAAAA |
| WF_core_107 | AGAGTTGGTAGCTCTAACTACGGCTA |
| WF_core_108 | CTCTGCTGAAGCCAGTCTGTCCGCCTTTCTCC |
| WF_core_109 | CTTCGGGAAGCGTGGCGTATTTGGTATCTGCG |
| WF_core_110 | CACTAGAAGAACAAACAGGATTAGCA |
| WF_core_111 | GAGCGAGGTATGTCCGGTAACTATCG |
| WF_core_112 | CCACTGGCAGCAGCCACTGGTGCTTTCTCATAGCTCACGCTGT |
| WF_core_113 | AGGTATCTCAGTTCGGTGTAGCCGGTAAGACACGACTTATCG |
| WF_core_114 | TCTTGAGTCCAACGTCGTTCGCTCCAAGC |
| WF_core_115 | TGGGCTGTGTGCACGAAGGAGGTTGAGGCAGG |
| WF_core_116 | GAAAATCTCCAAAAAAAAGAGTGAGAATAGAAAGGAA |
| WF_core_117 | TGTCGTCTTTCCAGACATAATAATTTTTTCACGTT |
| WF_core_118 | CAACTAAAGGAATTGCGAGTTAGTAAATGAA |
| WF_core_119 | CGGGGTTTTGCTCAGTACCCGCCACCCTCAGAACCGCCA |
| WF_core_120 | GCCACCCTCAGAACAGGCGGATAAGTGCCGTC |
| WF_core_121 | ATGCCCCCTGCCTATTATAGCCCGGAATAGGT |
| WF_core_122 | GAGAGGGTTGATATAAGTAGAGAAGGATTAGGATTAG |
| WF_core_123 | CTGAGACTCCTCATCGGAACCTATTA |
| WF_core_124 | GGAAAGCGCAGTCAAGTATTAAGAGG |
| WF_core_125 | TTCTGAAACATGAATAAGTTTTAACGGGGTCA |
| WF_core_126 | GAGTGTACTGGTATCTGAATTTACCG |
| WF_core_127 | GAATGGATCCTCACATACA |
| WF_core_128 | TTCCAGTAAGCGTTTAAAGCCAGAAT |
| WF_core_129 | ATACACAGAGTTATCGGATAGAACTTCT |
| WF_core_130 | ACTCGCGATAACCGTGTAGTAATTTATTT |
| WF_core_131 | CGTCACCTGGAGACGACGGGGGATTCA |
| WF_core_132 | ACGAAAACTTAAAGCAGACGAAGGGAAGAAAG |
| WF_core_133 | TTCCCCGAAAAGGTCGAGGACGACTACGGTCT |
|  |  |
| WF_FasL_handle_01 | TATTTTAATTCTAGGCGCCACGGCA TTTTCATTCTCCTATTACTACC |
| WF_FasL_handle_02 | AACCCAAAAGAACTCTCAGATACGTG TTTTCATTCTCCTATTACTACC |
| WF_FasL_handle_03 | AGTTTACAAGGAGCCCAGCATTGGCTACGCTAAG TTTTCATTCTCCTATTACTACC |
| WF_FasL_handle_04 | TTCCTTAGCTCCTGAAGCTATCCTAACGCA TTTTCATTCTCCTATTACTACC |
| WF_FasL_handle_05 | GCGCAGTTTTTCCGTTCCGCGCACA TTTTTCATTCTCCTATTACTACC |
| WF_FasL_handle_06 | GCAAGTGTAGCGGCATCCAGCAACGG TTTTCATTCTCCTATTACTACC |
| WF_biotin_handle_01 | TATTTTAATTCTAGGCGCCACGGCA TT [biotin] |
| WF_biotin_handle_02 | AACCCAAAAGAACTCTCAGATACGTG TT [biotin] |
| WF_biotin_handle_03 | AGTTTACAAGGAGCCCAGCATTGGCTACGCTAAG TT [biotin] |
| WF_biotin_handle_04 | TTCCTTAGCTCCTGAAGCTATCCTAACGCA TT [biotin] |
| WF_biotin_handle_05 | GCGCAGTTTTTCCGTTCCGCGCACA TT [biotin] |
| WF_biotin_handle_06 | GCAAGTGTAGCGGCATCCAGCAACGG TT [biotin] |
| WF_FISH_handle_01 | TATTTTAATTCTAGGCGCCACGGCA GCATTCTTTCTTGAGGAGGGCAGCAAACGGGAAGAG |
| WF_FISH_handle_02 | AACCCAAAAGAACTCTCAGATACGTG GCATTCTTTCTTGAGGAGGGCAGCAAACGGGAAGAG |
| WF_FISH_handle_03 | AGTTTACAAGGAGCCCAGCATTGGCTACGCTAAG GCATTCTTTCTTGAGGAGGGCAGCAAACGGGAAGAG |
| WF_FISH_handle_04 | TTCCTTAGCTCCTGAAGCTATCCTAACGCA GCATTCTTTCTTGAGGAGGGCAGCAAACGGGAAGAG |
| WF_FISH_handle_05 | GCGCAGTTTTTCCGTTCCGCGCACA GCATTCTTTCTTGAGGAGGGCAGCAAACGGGAAGAG |
| WF_FISH_handle_06 | GCAAGTGTAGCGGCATCCAGCAACGG GCATTCTTTCTTGAGGAGGGCAGCAAACGGGAAGAG |

**References**

1. Shang, X.; Bartels, N.; Weck, J. M.; Suppmann, S.; Basquin, J.; Heuer-Jungemann, A.; Monzel, C., *bioRxiv* **2024**.

2. Berger, R. M. L.; Weck, J. M.; Kempe, S. M.; Hill, O.; Liedl, T.; Radler, J. O.; Monzel, C.; Heuer-Jungemann, A., *Small* **2021,** *17* (26), e2101678. DOI 10.1002/smll.202101678.

3. Vanamee, E. S.; Faustman, D. L., *Sci Signal* **2018,** *11* (511). DOI 10.1126/scisignal.aao4910.

4. Gulculer Balta, G. S.; Monzel, C.; Kleber, S.; Beaudouin, J.; Balta, E.; Kaindl, T.; Chen, S.; Gao, L.; Thiemann, M.; Wirtz, C. R.; Samstag, Y.; Tanaka, M.; Martin-Villalba, A., *Cell Rep* **2019,** *29* (8), 2295-2306 e6. DOI 10.1016/j.celrep.2019.10.054.

5. Kaufmann, T.; Strasser, A.; Jost, P. J., *Cell Death Differ* **2012,** *19* (1), 42-50. DOI 10.1038/cdd.2011.121.

6. Ahn, J. H.; Park, S. M.; Cho, H. S.; Lee, M. S.; Yoon, J. B.; Vilcek, J.; Lee, T. H., *J Biol Chem* **2001,** *276* (50), 47100-6. DOI 10.1074/jbc.M107385200.

7. Wajant, H.; Pfizenmaier, K.; Scheurich, P., *Cytokine Growth Factor Rev* **2003,** *14* (1), 53-66. DOI 10.1016/s1359-6101(02)00072-2.
